# Loss of *RXFP2* and *INSL3* genes in Afrotheria shows that testicular descent is the ancestral condition in placental mammals

**DOI:** 10.1371/journal.pbio.2005293

**Published:** 2018-06-28

**Authors:** Virag Sharma, Thomas Lehmann, Heiko Stuckas, Liane Funke, Michael Hiller

**Affiliations:** 1 Max Planck Institute of Molecular Cell Biology and Genetics, Dresden, Germany; 2 Max Planck Institute for the Physics of Complex Systems, Dresden, Germany; 3 Center for Systems Biology Dresden, Germany; 4 Senckenberg Research Institute and Natural History Museum Frankfurt, Frankfurt am Main, Germany; 5 Museum of Zoology, Senckenberg Dresden, Germany; University of Bath, United Kingdom of Great Britain and Northern Ireland

## Abstract

Descent of testes from a position near the kidneys into the lower abdomen or into the scrotum is an important developmental process that occurs in all placental mammals, with the exception of five afrotherian lineages. Since soft-tissue structures like testes are not preserved in the fossil record and since key parts of the placental mammal phylogeny remain controversial, it has been debated whether testicular descent is the ancestral or derived condition in placental mammals. To resolve this debate, we used genomic data of 71 mammalian species and analyzed the evolution of two key genes (relaxin/insulin-like family peptide receptor 2 [*RXFP2*] and insulin-like 3 [*INSL3*]) that induce the development of the gubernaculum, the ligament that is crucial for testicular descent. We show that both *RXFP2* and *INSL3* are lost or nonfunctional exclusively in four afrotherians (tenrec, cape elephant shrew, cape golden mole, and manatee) that completely lack testicular descent. The presence of remnants of once functional orthologs of both genes in these afrotherian species shows that these gene losses happened after the split from the placental mammal ancestor. These “molecular vestiges” provide strong evidence that testicular descent is the ancestral condition, irrespective of persisting phylogenetic discrepancies. Furthermore, the absence of shared gene-inactivating mutations and our estimates that the loss of *RXFP2* happened at different time points strongly suggest that testicular descent was lost independently in Afrotheria. Our results provide a molecular mechanism that explains the loss of testicular descent in afrotherians and, more generally, highlight how molecular vestiges can provide insights into the evolution of soft-tissue characters.

## Author summary

While fossils of whales with legs demonstrate that these species evolved from legged ancestors, the ancestral state of nonfossilizing soft-tissue structures can only be indirectly inferred. This difficulty is also confounded by uncertainties in the phylogenetic relationships between the animals concerned. A prime example is the case of testicular descent, a developmental process that determines the final position of testes, which occurs in most placental mammals but is absent from several afrotherian lineages. Here, we discovered that afrotherians possess remnants of genes known to be required for testicular descent. These “molecular vestiges” show that testicular descent was already present in the placental ancestor and was subsequently lost in Afrotheria. Our study highlights the potential of molecular vestiges in resolving contradictory ancestral states of soft-tissue characters.

## Introduction

In placental mammals—the eutherian crown group consisting of the clades Afrotheria, Xenarthra, and Boreoeutheria [1]—optimal testicular function requires a temperature that is lower than the body temperature. To achieve this, the testes are located outside of the abdominal cavity in a scrotum in many species such as primates, most rodents, lagomorphs, most carnivores, and most terrestrial artiodactyls [2, 3]. Alternatively, testes are located in the lower abdomen in dolphins, true seals, pangolins, and other mammals. In these species, testicular cooling is achieved by vascular countercurrent heat exchanger systems, as observed in dolphin [4]; direct cooling with blood from the hind limbs, as observed in seals [5]; or testicular cooling may not be necessary, as these species have lower body temperatures [2, 6, 7].

The position of the testes in the lower abdomen or in the scrotum is the result of a developmental descent process (S1 Fig). During mammalian development, testes initially form at a position near the kidneys in the embryo. Testicular descent into the scrotum occurs in two phases: first from the abdomen to the inguinal canal and second through the inguinal canal into the scrotum [8, 9]. The first transabdominal phase is governed by the growth and reorganization of the gubernaculum, a ligament that connects the lower pole of the testes and inner ring of the future inguinal canal [8–10]. Migration of the testes is caused by the swelling of the distal gubernaculum, which anchors the testis to the inguinal canal, while the abdominal cavity enlarges. The second inguinoscrotal phase is dependent on androgen signaling and requires the elongation of the gubernaculum, which migrates into the scrotum [8–10]. The involved signaling and mechanics make testicular descent a difficult and complex developmental process. Failure in any of the descent phases results in a pathological condition called cryptorchidism (absence of testes from the scrotum), which is a congenital birth defect observed at an appreciable frequency in human males (2%–4% at birth [11]) and other animals (up to 10% in male dogs [12], 2% in male cats [13], 2%–8% in male horses [14]).

Almost all placental mammals exhibit either partial descent (only the transabdominal phase), which results in ascrotal testes located in the lower abdomen, or complete descent (transabdominal and inguinoscrotal phase), which results in scrotal testes [2, 3]. A notable exception is Afrotheria, in which five of the six main lineages (represented here by the lesser hedgehog tenrec, cape golden mole, cape elephant shrew, manatee, elephant, and rock hyrax) do not show any testicular descent and have testes positioned at their initial abdominal position near the kidneys [2, 3, 15–17]. This lack of any testicular descent is termed testicondy. The aardvark is the only afrotherian exhibiting descended but ascrotal testes [2, 3, 18]. A schematic illustration of the different position of testes in mammals is shown in S1 Fig.

Since Afrotheria represent one of the three main clades of placental mammals (together with Xenarthra and Boreoeutheria), two different evolutionary scenarios could explain testicondy in several afrotherian lineages. First, if testicondy is the ancestral condition in placental mammals, then testicular descent was gained two or three times (depending on the phylogeny) in Xenarthra, Boreoeutheria, and the aardvark lineage. Second, if testicular descent is the ancestral condition in placental mammals, then testicular descent was lost once or more often (again depending on the phylogeny) in five of the six afrotherian lineages. Since soft-tissue structures like testes and the transient gubernaculum ligament are typically not preserved in the fossil record, the evolution of such soft-tissue structures can only be inferred by analytical methods such as parsimony, extant phylogenetic bracketing, or maximum likelihood [19–22], all of which rely on the given phylogenetic tree. Consequently, resolving whether testicondy or testicular descent is the ancestral condition in placental mammals requires accurate knowledge of the underlying phylogeny.

Unfortunately, although integrative approaches using both morphological and molecular characters have brought major advances in our understanding of mammalian phylogeny [23–26], there is still no final consensus on the relationships between (and sometimes within) the main clades of placental mammals. In particular, the placental root and branching pattern of the clades Afrotheria, Xenarthra, and Boreoeutheria are still debated [26–28] (S2 Fig), and an analysis of rare genomic events raised the concrete possibility of a near-simultaneous split [29]. Furthermore, the phylogeny within Afrotheria is not well resolved, because of conflicting evidence for the position of the aardvark (the only nontesticond afrotherian lineage) and the relationships between manatees, elephants, and hyraxes [30–34] (S3 Fig).

Given these phylogenetic uncertainties, it is probably not surprising that two different studies reached opposite conclusions about whether testicondy is the ancestral or derived state for placental mammals and for Afrotheria. Werdelin and Nilsonne [2] inferred that testicular descent in placental mammals and Afrotheria is the ancestral condition (testicular descent was subsequently lost). However, their results were based on a phylogeny in which Afrotheria were nested within Boreoeutheria, which is not supported by current phylogenies. More recently, Kleisner and colleagues [3] reexamined the evolution of testicular descent in the context of current phylogenies and came to the opposite conclusion that testicondy in placental mammals and Afrotheria is the ancestral phenotypic character.

Here, we sought to resolve this debate whether testicondy or testicular descent is the ancestral condition in placental mammals and in Afrotheria by using molecular evidence. First, we reasoned that if testicular descent is ancestral, then testicond afrotherian lineages may have lost key genetic information that is necessary for testicular descent. Such a loss of genetic information may be detectable by comparative genomics analysis. Second, we reasoned that if testicular descent is ancestral and if aardvarks are nested within Afrotheria, then testicondy would have evolved independently several times. This is expected to leave a signature of independent loss of the genetic information that is necessary for testicular descent. By analyzing the evolution of two key genes (relaxin/insulin-like family peptide receptor 2 [*RXFP2*] and insulin-like 3 [*INSL3*]) that are required for gubernaculum development and function in 71 placental mammals, we found that both genes have loss-of-function mutations only in several testicond afrotherian species. The absence of shared inactivating mutations and our age estimates for the loss of *RXFP2* further suggest that testicondy evolved independently in afrotherian lineages at different time points. Together, these results provide not only a molecular mechanism that explains the loss of testicular descent in afrotherian lineages but also shows that testicular descent is the ancestral state for placental mammals and Afrotheria.

## Results

### Comparative analysis of the gubernaculum-inducing *RXFP2* and *INSL3* genes in 71 placental mammals

To determine if testicondy is the ancestral or derived condition for placental mammals and for Afrotheria, we examined two key genes that are necessary and sufficient for the development of the gubernaculum: *INSL3* and *RXFP2*. *INSL3* encodes a relaxin-like hormone that is secreted by Leydig cells of the testes and binds specifically to the transmembrane receptor encoded by *RXFP2*, which is highly expressed in gubernacular cells [35–39]. The *INSL3*-*RXFP2* ligand-receptor pair promotes gubernacular cell proliferation and stimulates the swelling reaction [40–42]. Both genes are necessary for gubernacular function, as knockout of *RXFP2* [37, 39] or *INSL3* [40, 43, 44] in mice results in the absence of the gubernaculum and no testicular descent, which in turn leads to spermatogenesis defects and male infertility. Despite the fact that *RXFP2* is also expressed in postmeiotic spermatogenic cells, surgically correcting the position of undescended testes in global *INSL3* knockout mice or a knockout of *RXFP2* that is restricted to male sperm cells results in normal spermatogenesis and fertility [39, 43], suggesting that both genes are dispensable for spermatogenesis and germ cell survival in adult male mice.

To investigate the evolution of *RXFP2* and *INSL3* in placental mammals, we made use of existing genome alignments between humans and 68 other mammals [45]. In addition, we further computed a genome alignment between human and the most recent genome assemblies of the rock hyrax and Hoffmann’s two-toed sloth (Materials and methods). Inspecting the genomic loci that correspond to human *RXFP2* and *INSL3* allowed us to examine both genes in all 70 mammals, even in the absence of gene annotations for most of these species.

### Loss of the *RXFP2* and *INSL3* genes in four testicond afrotherian lineages

**Fig 1:**
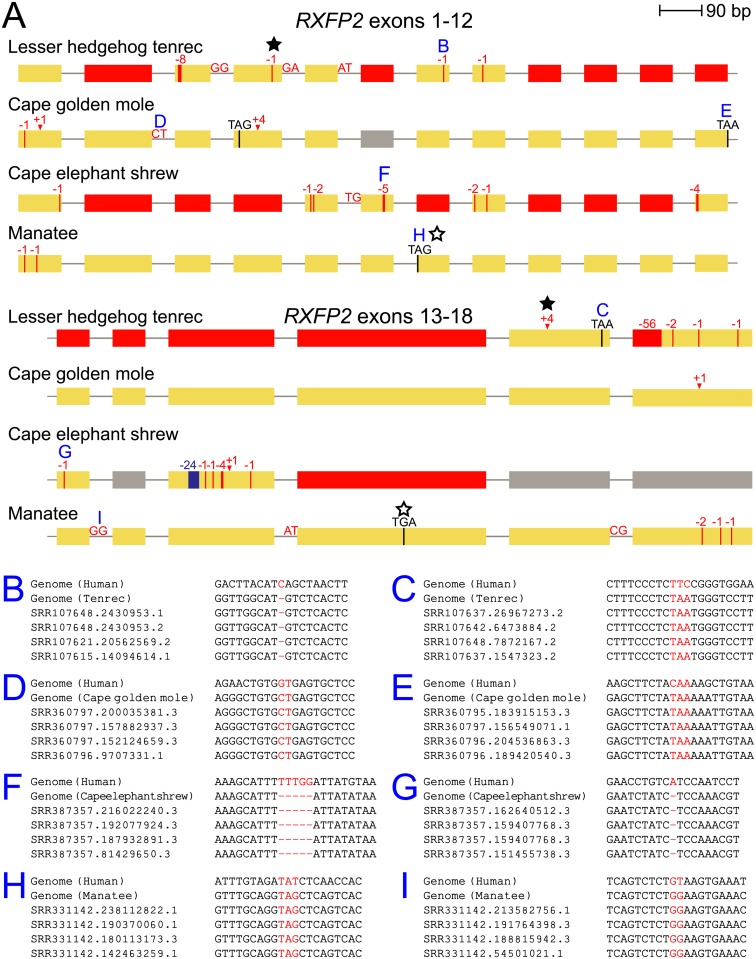
Gene-inactivating mutations in *RXFP2* in four afrotherian species. (A) The exon-intron structure of the coding region of the *RXFP2* gene is shown as boxes (exons, drawn to scale) and lines (introns, not drawn to scale). A vertical red line/arrowhead indicates a frameshifting deletion/insertion, with the number of deleted/inserted bases given above. Stop codon mutations are shown as a black vertical line. Splice site mutations are indicated by the mutated dinucleotide. A blue vertical line indicates a frame-preserving deletion. Red boxes are exons that are either deleted or accumulated numerous mutations that destroy any sequence similarity. All inactivating mutations were validated by unassembled genome sequencing reads stored in the SRA. Elephant, rock hyrax, and aardvark have an intact *RXFP2* gene and are not shown. A filled star indicates mutations that we confirmed by PCR and Sanger sequencing in the lesser hedgehog tenrec; the exon 17 frameshift was also found in the greater hedgehog tenrec (S5A and S5B Fig). An open star indicates mutations that we confirmed by PCR and sequencing in the dugong, the sister species of the manatee (S5C and S5D Fig). (B-I) Examples of inactivating mutations and their validation by unassembled SRA reads. *RXFP2*, relaxin/insulin-like family peptide receptor 2; SRA, Sequence Read Archive.

**Fig 2:**
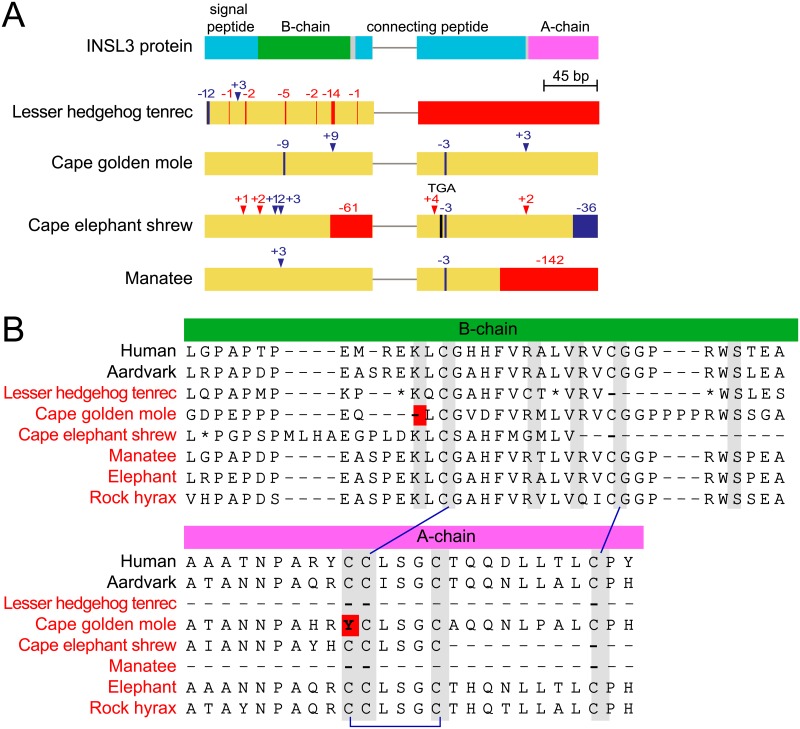
Gene-inactivating mutations and critical amino acid mutations in *INSL3*. (A) Functional domains of the INSL3 protein and the exon-intron structure of the *INSL3* gene. Inactivating mutations are as in Fig 1, frame-preserving insertions/deletions are shown as blue lines/arrowheads. Exons but not introns are drawn to scale. Elephant, rock hyrax, and aardvark have an intact *INSL3* and are not shown. (B) While the cape golden mole does not exhibit any gene-inactivating mutations (A), an INSL3 protein alignment of the A- and B-chain shows mutations (red background) at amino acids that are critical for structure and function of the mature hormone (gray background) [46–48]. Disulfide bonds between Cys residues are indicated by blue lines. Residues that are affected by frameshifting deletions in the underlying tenrec or cape elephant shrew nucleotide sequence are indicated by asterisks. For these two species, we ignored these frameshifts and used the ancestral reading frame. Species in red font are testicond. Note that elephant and rock hyrax have no mutations at any of the critical sites. *INSL3*, insulin-like 3.

To investigate if testicond Afrotheria lost the genetic information necessary for testicular descent, we first examined the coding region of *RXFP2* and *INSL3* in seven afrotherians with available genomes (aardvark, lesser hedgehog tenrec, cape golden mole, cape elephant shrew, manatee, elephant, and hyrax). Our genome alignments revealed that four testicond lineages (tenrec, cape golden mole, cape elephant shrew, and manatee) have several mutations in *RXFP2* that inactivate its reading frame. These gene-inactivating mutations create premature stop codons, shift the reading frame, disrupt the splice site dinucleotides, and delete entire exons (Fig 1A). Furthermore, three out of these four species (tenrec, cape elephant shrew, manatee) also have inactivating mutations in the *INSL3* gene (Fig 2A). Since these mutations affect several exons and destroy functional protein domains in INSL3 (A- and B-chain, Fig 2A), it is highly unlikely that the remnants of these genes encode a functional protein. Importantly, reciprocal-best BLAST hits and conserved gene order clearly show that these remnants are “molecular vestiges” that correspond to the *RXFP2* and *INSL3* genes (S4 Fig). In analogy to vestigial organs, these molecular vestiges imply the presence of once functional *RXFP2* and *INSL3* orthologs that were subsequently lost in several afrotherians during evolution.

To confirm that these inactivating mutations are real and do not represent genome assembly or alignment errors, we used a multistep validation approach. Since genome alignments do not take reading frame and splice site information into account, we first sought to rule out the possibility that inactivating mutations are a consequence of alignment ambiguities. To this end, we realigned all coding exons with the Coding Exon-Structure Aware Realigner (CESAR), an exon alignment method that produces an alignment with consensus splice sites and an intact reading frame whenever possible [49, 50]. CESAR confirmed that all affected exons exhibit inactivating mutations (Figs 1 and 2A). Second, to validate that these mutations are not sequencing or assembly errors, we investigated raw sequencing reads from the Sequence Read Archive (SRA) [51]. For both *RXFP2* and *INSL3*, we found that all genomic loci containing an inactivating mutation are supported by at least 10 sequencing reads, while not a single read aligns to a putative sequence, in which the inactivating mutation was reversed to its ancestral state. We further confirmed the presence of two frameshifting mutations in *RXFP2* in the lesser hedgehog tenrec by PCR and Sanger sequencing (S5A and S5B Fig). Overall, this shows that the inactivating mutations shown in Figs 1 and 2A are not sequencing errors or artefacts arising from genome assembly or alignment issues. Finally, we investigated whether hitherto undetected functional copies of *RXFP2* or *INSL3* exist in afrotherians that may have arisen by lineage-specific duplications. By performing ultra-sensitive genome alignments, we only detected a single orthologous locus for *RXFP2* and *INSL3*. In addition, we found alignments to *RXFP1*, a paralog of *RXFP2* that exists in all placental mammals (S6 Fig), showing that these alignment parameters are sufficiently sensitive to even detect more ancient gene duplications. Together, this excludes the possibility that afrotherians possess another functional duplicated copy of *RXFP2* or *INSL3*.

If *RXFP2* and *INSL3* are truly lost, we further expect that they evolve neutrally in the lineages with inactivating mutations. Indeed, using RELAX [52], we found that *RXFP2* evolves under relaxed selection in all four gene-loss species (adjusted *P* values < 3.2e^−5^, S1 Table). For *INSL3*, no significant evidence for relaxed selection was found, likely because large deletions in this short 131-residue protein in tenrec, cape elephant shrew, and manatee (Fig 2A) severely reduced alignment length. Therefore, we inspected the two protein domains that are necessary for the function of the mature INSL3 hormone. Similar to insulin, the preprohormone INSL3 is processed into an A- and B-chain peptide. The A- and B-chain then forms a heterodimer that is stabilized by two disulfide bonds between the A- and B-chains and one disulfide bond within the A-chain [47]. We found that tenrec, cape elephant shrew, and manatee have deletions that overlap the A- and B-chain and affect residues that are critical for INSL3 structure and function (Fig 2B). Together, our results conclusively show that the remnants of *RXFP2* and *INSL3* cannot encode functional proteins in several testicond afrotherian lineages.

Since *INSL3* lacks clear inactivating mutations in the cape golden mole, we examined the residues that are important for INSL3 structure and function. We found that the Cys at position 10 in the A-chain that forms a disulfide bond with Cys at position 15 [47] is mutated to a Tyr in the cape golden mole (Fig 2B). Furthermore, the Lys at position 8 in the B-chain (Fig 2B), a residue that is important for receptor activation [48], is deleted in this species. This suggests that, while *INSL3* still has an intact reading frame in the cape golden mole, it accumulated mutations that most likely render the encoded protein nonfunctional.

### *RXFP2* and *INSL3* are intact in elephant and rock hyrax

Interestingly, both *RXFP2* and *INSL3* lack any gene-inactivating mutations in the elephant and the rock hyrax, two afrotherians that are also testicond [15, 17]. While the elephant has a 2-bp deletion in the last exon of *RXFP2*, this merely truncates the C-terminus by 25 residues and is not an indication of loss (see section *RXFP2* and *INSL3* are intact in all nontesticond placental mammals and S7 Fig). Furthermore, RELAX estimates a Ka/Ks value of 0.31 and 0.33 for elephant and rock hyrax, respectively, which is slightly but not significantly higher than the Ka/Ks value of 0.27 observed for other mammals. Thus, there is no significant evidence for relaxed selection in these two lineages. We also scanned both genes for amino acid mutations that were only observed in human cryptorchidism patients (V18M, P49S, W69R, P93L, R102C, R102H, R105H, N110K in INSL3 and T222P in RXFP2 [8]). Whereas elephant INSL3 exhibits the R102H mutation, this mutation is observed in many other nontesticond mammals, and cell line experiments have shown that this mutation does not affect INSL3 activity [38]. Similarly, elephant RXFP2 has a T222A (Thr to Ala) mutation at a position where a mutation from Thr to Pro renders RXFP2 nonfunctional [37, 53]. However, the T222A mutation that is present in elephant is also observed in the nontesticond aardvark and pangolin, and experiments have shown that mutating this Thr to Ala does not affect RXFP2 function [53]. The rock hyrax does not exhibit any of the mutations observed in human cryptorchidism patients. Based on these evidences, elephant and rock hyrax *RXFP2* and *INSL3* may encode functional proteins.

### *RXFP2* and *INSL3* are intact in all nontesticond placental mammals

So far, our analysis suggests that the function of *RXFP2* and *INSL3* is only compromised in several testicond afrotherians. Therefore, we examined both genes in the aardvark, the only afrotherian exhibiting partial testicular descent [2, 18], and found that *RXFP2* and *INSL3* are intact and evolve under selection (S1 Table) and that INSL3 lacks any mutations of critical amino acids (Fig 2B). To further investigate the relation between loss of *RXFP2* and *INSL3* and testicondy, we examined both genes in 64 other nontesticond mammals. While the genome alignment showed a few putative inactivating mutations in some species, a detailed manual inspection revealed that these are assembly errors (S8 Fig), assembly gaps (S9 Fig), and alignment ambiguities (S10 Fig). For *RXFP2*, we further found that the N-terminus is 17 amino acids longer in human, chimpanzee, bonobo and gorilla (S11 Fig) and that several species have small truncations and elongations of the C-terminus without affecting the transmembrane domains of the receptor (S7 Fig). Since *RXFP2* does not evolve under relaxed selection in any of these 64 mammals (S1 Table), these length variations are not an indication of gene loss but support previous observations that N- and C-termini of proteins are evolutionarily less constrained [49, 54]. Together, this shows that both genes are intact and under selection in all other nontesticond placental mammals.

### Dating the gene loss events indicates that testicondy evolved independently in Afrotheria

**Fig 3:**
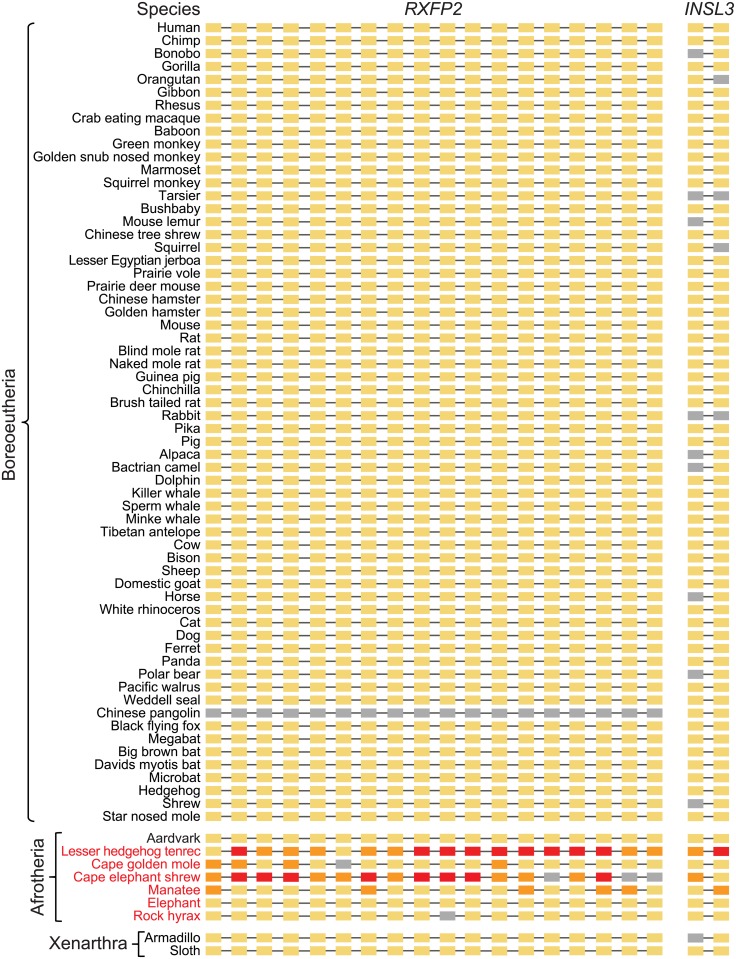
*RXFP2* and *INSL3* are only lost in several testicond afrotherian lineages. The exon-intron structures of *RXFP2* (18 coding exons) and *INSL3* (2 coding exons) are shown. A yellow rectangle indicates an intact exon. Exons with inactivating mutations (stop codon or splice site mutations, frameshifts) are indicated in orange; completely deleted or highly diverged exons are indicated in red. Gray exons could not be examined because the respective genome assembly is incomplete at this locus (assembly gap). We could not examine the *RXFP2* in the fragmented pangolin genome, because different parts of *RXFP2* locus align to at least 5 different short scaffolds. Neither exons nor introns are drawn to scale. *INSL3*, insulin-like 3; *RXFP2*, relaxin/insulin-like family peptide receptor 2.

Interestingly, we observed no inactivating mutations in *RXFP2* and *INSL3* that are shared among any testicond afrotherian species, suggesting that the loss of these genes happened independently, after these species split from their common ancestors. Since *RXFP2* and *INSL3* are expected to evolve neutrally after the loss of testicular descent, an estimate of how long these genes have been evolving neutrally provides an estimate for when testicondy occurred. To this end, for the four branches in the phylogenetic tree leading to the four gene-loss species, we estimated the portion of the branch where the gene evolved under selection and the portion where it evolved neutrally, as described in [55, 56]. Since large parts of *INSL3* are deleted in tenrec, cape elephant shrew and manatee (Fig 2A) and since exon 1 overlaps assembly gaps in several other species (Fig 3), we focused on *RXFP2*, for which each gene-loss species provides at least 594 bp in the codon alignment, to obtain robust estimates.

**Fig 4:**
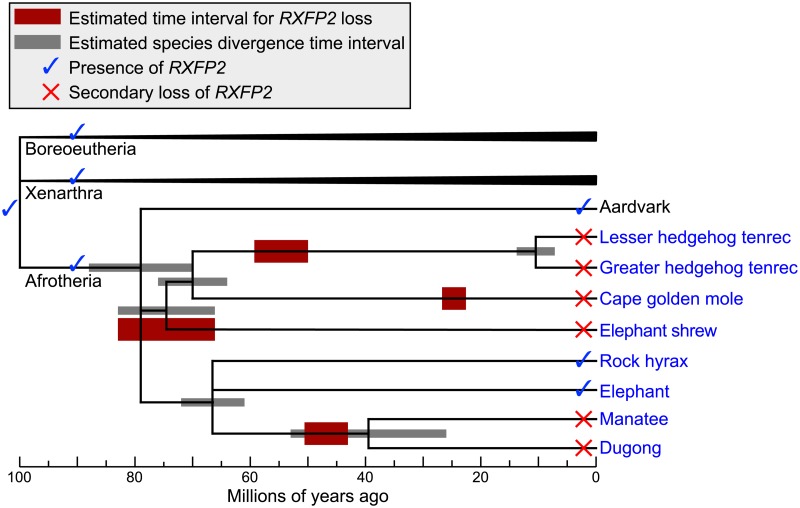
Evolution of *RXFP2* and estimated time intervals during which *RXFP2* came under neutral evolution. Red boxes represent a lower and upper bound for an estimated time interval during which *RXFP2* started to evolve neutrally (Materials and methods, S2 Table), which shows that the gene was lost independently at different time points. Gray boxes represent an estimated interval for species divergence times taken from TimeTree [57] (see also S4 Table). Species in blue font are testicond. The phylogenetic position of tenrecs, golden moles, elephant shrews is well supported by morphological and molecular characters [23, 24, 26]. Uncertain phylogenetic relationships are shown as a polytomy. Note that while the loss of *RXFP2* in the elephant shrew is estimated to have happened at the base of the lineage, this loss is clearly independent from the much later loss in the tenrec and in the golden mole lineage. Evidence for the loss of *RXFP2* in the dugong and the greater hedgehog tenrec was obtained by PCR and sequencing experiments (S5 Fig). *RXFP2*, relaxin/insulin-like family peptide receptor 2.

As shown in Fig 4 and S2 Table, we estimate that each lineage lost the *RXFP2* gene at different time points. Consistent with the large number of inactivating mutations, *RXFP2* appears to be lost first in the cape elephant shrew around 66–83 million years ago (Mya). For the lesser hedgehog tenrec, we estimate that *RXFP2* loss happened around 50–59 Mya. Consistent with this estimate, we found that the same gene-inactivating mutation in *RXFP2* exon 17 is shared with its sister species greater hedgehog tenrec (S5B Fig), suggesting that *RXFP2* loss already occurred in the ancestor of both tenrec species that lived 7–14 Mya (Fig 4). The loss of *RXFP2* in manatee is estimated to have happened around 43–51 Mya and thus likely predates the split of the manatee and dugong lineage 26–53 Mya. To test this, we used PCR and sequencing experiments and found that the dugong shares two stop codon mutations in different exons with the manatee (S5C and S5D Fig), confirming that *RXFP2* loss predates the split of manatees and dugongs. The loss in the cape golden mole likely happened more recently (23–28 Mya), consistent with our observations that *INSL3* did not yet accumulate a gene-inactivating mutation. While the absolute estimates of gene-loss times are tentative (as fossil-based time calibrations of the species divergence times are lacking), these time intervals are substantially different from each other (Fig 4). Together with the absence of shared inactivating mutations, this strongly suggests that testicondy evolved independently in Afrotheria.

## Discussion

The evolution of testicondy and whether testicular descent is the ancestral [2] or derived state [3] for placental mammals and for Afrotheria has been controversial, despite agreement in the phenotypic character assignment. The different conclusions are mainly due to persisting differences in the phylogeny, which affect ancestral character reconstruction. To resolve this debate, we investigated the evolution of *RXFP2* and *INSL3*, two genes encoding a hormone receptor pair that is required for the development of the testes-descending gubernaculum ligament. We found remnants of once functional orthologs of *RXFP2* and *INSL3* as molecular vestiges in four testicond afrotherian lineages. Together with the presence of orthologs of both genes in other Afrotheria and other placental mammals, this shows that these genes were lost after the testicond lineages split from the afrotherian ancestor. This allows us to conclude that testicular descent is the ancestral condition in placental mammals and was subsequently lost in different afrotherian lineages. Importantly, our conclusion holds regardless of persisting phylogenetic discrepancies that involve the branching pattern of Afrotheria, Xenarthra, and Boreoeutheria at the placental root, and the phylogeny within Afrotheria (S2 and S3 Figs).

Our study also provides three lines of evidence that testicondy evolved independently in Afrotheria. First, if testicondy evolved in the common ancestor of any two testicond afrotherians, we would expect inactivating mutations in *RXFP2* and *INSL3* that are shared among species. However, both genes do not exhibit any shared inactivating mutations. Second, by estimating how long these genes have been evolving neutrally, we found different time intervals in which gene loss and likely testicondy evolved. These estimated time intervals may be helpful to better understand the ecological conditions under which testicondy evolved repeatedly. Third, independent evolution of testicondy is further supported by our finding that the testicond elephant and rock hyrax have intact *RXFP2* and *INSL3* genes that still appear to evolve under purifying selection. Since recent shifts from purifying selection to neutral evolution will not leave a detectable signature of significantly increased Ka/Ks ratios, our results suggest that elephant and rock hyrax *RXFP2* and *INSL3* may have come under relaxed or no selection in recent evolutionary time. In agreement with a more recent evolution of testicondy, the rock hyrax still exhibits rudiments of gubernacular structures [15]. It is possible that amino acid mutations or *cis*-regulatory mutations that affect the expression of *RXFP2* or *INSL3* are responsible for testicondy in elephant and rock hyrax. Indeed, the *RXFP2* promoter region exhibits more sequence divergence in the rock hyrax than in all other nontesticond species, and the elephant sequence also shows elevated divergence compared to most but not all mammals (S12 Fig). However, the precise genomic basis of testicondy in elephant and rock hyrax remains to be elucidated.

Spermatogenesis and sperm storage require temperatures below 37 °C. Body temperatures below 35 °C, as observed in the heterothermic tenrecs and golden moles [7], may abolish the need for testicular cooling, which would explain why testicondy is tolerated in these species. However, other afrotherian species have body temperatures similar to many scrotal mammals. For example, the body temperature of elephants and elephant shrews is 36.8 °C and 37.2 °C, respectively [7, 58]. This raises the questions of how these testicond afrotherian lineages maintain normal testicular function at a body temperature of approximately 37 °C and whether these species have evolved novel cooling mechanisms. Previous anatomical studies did not reveal conclusive evidence of such cooling systems. In particular, testicond afrotherians do not possess a pampiniform plexus or a vascular countercurrent heat exchanger that could act as a comparable cooling system [17, 59, 60]. In the dugong, the epididymis has a large, folded surface and is surrounded by highly vascularized tissue [60]; however, whether this arrangement acts as a cooling system is not clear. In rock hyrax and elephant, it was observed that the part of the testicular duct that stores spermatozoa lies close to the body surface [59], but it remains unknown whether this anatomical feature has a cooling function during sperm storage. Hence, detailed comparative and functional studies of the testes, epididymis, and their associated anatomical structures are required to understand whether Afrotheria evolved novel mechanisms for sperm cooling during spermatogenesis and storage. Furthermore, it would be of great interest to learn why several afrotherian but not any other placental lineages have completely lost testicular descent. For example, it is unknown whether the loss of testicular descent is beneficial for these species, whether anatomical constraints associated with body plan or life history explain the loss of descent, or whether this phenotypic reversal is an evolutionary tradeoff for another advantageous trait. More anatomical studies that in particular compare the development of Afrotheria are required to address these questions.

More generally, our results highlight how molecular evolution can shed light on the evolution of phenotypes. While the fossil record has contributed substantially to our understanding of how hard-tissue characters such as bones, teeth, or shells evolved, the evolution of soft-tissue structures that do not fossilize can often only be inferred with analytical methods. Moreover, conclusions about the ancestry of such characters are dependent on the underlying phylogeny. Molecular vestiges offer an alternative strategy to investigate character ancestry. This strategy may be broadly applicable, since molecular vestiges are also known for other phenotypes whose ancestry is not controversial based on fossil evidence and accurate knowledge of the underlying phylogeny. For example, remnants of enamel-related genes in toothless mammals [61–63], remnants of hair development genes in hairless cetaceans [63], remnants of gastric genes in vertebrates without a stomach [64], or remnants of eye-related genes in subterranean mammals with degenerated eyes [65–68] show that these species descended from ancestors with teeth, hair, a stomach, and functional eyes, respectively. Thus, instead of investigating a soft-tissue structure directly, one can trace the evolution of genes that are crucial for the development of this structure. Molecular vestiges of such genes can then provide insights into the ancestry of soft-tissue structures, even if the phylogenetic positions of the respective species remain controversial.

## Materials and methods

### Genome alignments

To investigate the coding region of *RXFP2* and *INSL3* in placental mammals, we used an alignment between the human hg38 genome assembly and the genomes of 68 nonhuman placental mammals [45]. Since this alignment does not provide the rock hyrax (*Procavia capensis*) and Hoffmann’s two-toed sloth (*Choloepus hoffmanni*), two mammals for which improved genome assemblies have recently become available, we used the pipeline of lastz (version 1.03.54, parameters K = 2,400, L = 3,000, Y = 9,400, H = 2,000, and the lastz default scoring matrix) [69], chaining (parameters chainMinScore 1,000, chainLinearGap loose), and netting (default parameters) [70] to compute new genome alignments. Since several species have assembly gaps overlapping *INSL3* exon 1, likely because this GC-rich region gets poor coverage in Illumina sequencing, we further computed genome alignments using updated assemblies of bonobo, domestic goat, camel, and dolphin. We also inspected the genome alignment to the recent gorGor5 assembly of the gorilla, provided by UCSC [71]. For gorilla, goat, and dolphin, the new assemblies closed these assembly gaps and revealed an intact *INSL3* exon 1. For bonobo and camel, the new assemblies still have assembly gaps overlapping *INSL3* exon 1. S3 Table lists all species and their assemblies that were analyzed.

We examined the colinear alignment chains (loaded in the UCSC genome browser) in order to assess if gene order around the *RXFP2* and the *INSL3* genes is conserved in Afrotheria. This showed that both genes and several neighboring genes occur in a conserved order in afrotherian genomes. Furthermore, existing gene annotations (Ensembl) and reciprocal-best BLAST of the human proteins confirmed that the aligning loci contain orthologous *RXFP2* and *INSL3* genes or their remnants.

To perform ultra-sensitive genome alignments that could reveal any hitherto undetected functional copies of *RXFP2* or *INSL3*, we first computed genome alignments between the human genome (hg38 assembly) and each of the seven afrotherians, using highly-sensitive lastz parameters by setting K = 2,000 and L = 2,500. Subsequently, we used even more sensitive parameters to find additional local alignments by running lastz with parameters K = 1,500, L = 2,200, and W = 5 on all chain gaps (nonaligning regions flanked by aligning blocks) that are at least 30 bp and at most 500 Kb long, as described in [45, 72]. We built alignment chains from all local alignments [70] and visualized them in the UCSC genome browser [71].

### Investigating *RXFP2* and *INSL3* in 70 nonhuman mammals

We used the genome alignments to systematically search each exon for frameshifting insertions and deletions, mutations that create in-frame stop codons, and mutations that disrupt the conserved splice site dinucleotides (donor GT/GC, acceptor AG). We validated each putative mutation by the following steps: First, we realigned the exonic sequence of each exon with an inactivating mutation, using CESAR [49, 50] with default parameters. CESAR is a Hidden-Markov-Model based aligner that takes the reading frame and the splice sites into account and tries to produce an intact exon alignment (no inactivating mutations, consensus splice sites) between human and a query species, wherever possible. All exons for which CESAR confirmed the existence of at least one inactivating mutation are shown in Fig 3. S10 Fig shows an example for which CESAR found an alternative alignment that lacks inactivating mutations.

Second, we validated the remaining inactivating mutations by searching SRA for unassembled sequencing reads that have a 100% match to the genomic context comprising at least 50 bp around the mutation, as described in [73]. Furthermore, we searched SRA for a putative sequence, in which the inactivating mutation was reversed to its ancestral state. Every mutation that is supported by at least 10 sequencing reads without any hit to the putative “ancestralized” sequence was considered as real.

Third, we examined all exons that do not align in the query genome, since these are either truly deleted or an artifact arising from incomplete genome assemblies [68, 74]. We used the nearest up- and downstream aligning blocks in the alignment chain to determine the genomic locus that corresponds to this exon in the query genome. If this locus does not overlap an assembly gap, we concluded that the exon is lost (either deleted or accumulated so many mutations that it does not align anymore). If this locus overlaps an assembly gap, we conservatively considered this exon as missing sequence, as described in [63]. Indeed, as shown in S9 Fig, exons that overlapped assembly gaps in a previous assembly readily aligned with an intact reading frame in an improved genome assembly, showing that assembly gaps should not be mistaken for exon loss.

### Determining if the genes are under relaxation in different species and the associated Ka/Ks values

To determine if the *RXFP2* coding sequence evolves neutrally in different species, we used RELAX version 2 [52]. Briefly, given an alignment, a tree topology, and branches labeled as either test or reference, RELAX determines if the gene evolves under relaxed or intensified selection in the test branches relative to the reference branches. We used RELAX’s partition descriptive method that fits three Ka/Ks classes to the test and the reference branches and also estimates an overall Ka/Ks value. To determine if *RXFP2* evolves under relaxed selection in any mammal, we iteratively applied RELAX, labeling each of the 71 species as the test branch. The resulting *P* values were corrected for multiple testing using the Benjamini and Hochberg method (S1 Table). To exclude any bias caused by relaxed selection in the *RXFP2*-loss species, which might inflate the reference Ka/Ks values, we further applied RELAX, labeling only one afrotherian species as test and excluding all other afrotherian species from the reference branches. These tests confirmed that *RXFP2* evolves under relaxed selection only in the four afrotherians with an inactivated *RXFP2* gene but not in the three other afrotherian species (S1 Table).

### Dating gene loss events

To date the loss of *RXFP2*, we followed the procedure described in [55, 56]. For a branch along which the gene was inactivated, this method assumes that a gene evolves under a selective pressure similar to that in other species until it is inactivated. Afterward, the gene is assumed to accumulate both synonymous and nonsynonymous mutations at a neutral rate. The Ka/Ks value (K) estimated for this entire branch is then the average of the Ka/Ks value for the part of the branch where the gene was under selection (K_s_) and the Ka/Ks value for the part of the branch where the gene evolved neutrally (K_n_ = 1), weighted by the proportion of time for which the gene was evolving under selection (T_s_ / T) and neutrally (T_n_ / T):

$$\text{K}={\text{K}}_{\text{s}}\times {\text{T}}_{\text{s}}/\text{T}+{\text{K}}_{\text{n}}\times {\text{T}}_{\text{n}}/\text{T},$$

where T represents the time since the split from the last common ancestor. Using the lower and upper bound of the confidence interval for the species divergence time T obtained from TimeTree [57] (S4 Table) and using the Ka/Ks value for mammals with a functional *RXFP2* (K_s_ = 0.2491), one can estimate a lower and upper bound for T_n_ as

$${\text{T}}_{\text{n}}=\text{T}\times (\text{K}-\;{\text{K}}_{\text{s}})\;/\;(1-{\text{K}}_{\text{s}}),$$

which provides an estimate of how long *RXFP2* has been evolving neutrally (S2 Table).

### PCR experiments

DNA of the greater hedgehog tenrec (*Setifer setosus*) and the lesser hedgehog tenrec (*Echinops telfairi*) was kindly provided by Athanasia Tzika (University of Geneva). Dugong tissue was kindly provided by Joerns Fickel (Leibniz-Institute of Zoo and Wildlife Research, Berlin). DNA was extracted using the innuPrep DNA Mini Kit (Analytik Jena, Jena, Germany) following the manufacturer’s instructions (protocol for DNA isolation from tissue samples or rodent tails) but extending tissue lysis overnight and including RNA digestion.

Standard PCR reactions were performed in a total volume of 10 μl containing 5–20 ng DNA, 5 μl Phusion Flash High-Fidelity PCR Master Mix (Thermo Scientific, Waltham, United States of America; #F-548S), 0.25% DMSO (New England Biolabs, Frankfurt Main, Germany), and 0.5 μM of each primer (S5 Table). Cycling conditions were as follows: 35 cycles were used with denaturation at 98 °C (15 s but 2 min for the first cycle), annealing at a primer pair–specific temperature (S5 Table, 20s), and extension at 72 °C (10 s–1 min but 5 min after the last cycle).

PCR products were Sanger-sequenced after enzymatic cleanup with CleanDTR (GC biotech, Waddinxveen, Netherlands) followed by cycle sequencing with the PCR primers and a mix of the BigDye Terminator v3.1 Cycle Sequencing Kit and dGTP BigDye Terminator v 3.0 Kit (Applied Biosystems, Foster City, CA, USA). Analyses were performed on an ABI 3730xl DNA Analyser (Applied Biosystems).

### Promoter sequence divergence

We analyzed per-species sequence divergence of the promoter region of *RXFP2* and *INSL3*. Using the human hg38 assembly coordinates chr13:31739464–31739544 and chr19:17821524–17821716, we extracted the sequence of all placental mammals, used PRANK [75] with parameters “-keep -showtree -showanc -prunetree” to align these sequences and to reconstruct the most likely sequence of the placental mammal ancestor and measured the percent sequence identity between the ancestral sequence and the sequence of every extant mammal, as described in [68, 74]. The result is visualized in S12 Fig.

## Supporting information

S1 Fig Illustration of the process of testicular descent and the position of testes in different mammals. (A) Illustration of the developmental process that results in testicular descent. The gubernaculum is shown in green. (B) Simplified representation of the position of the testes (orange) and kidneys (blue) illustrating the three conditions discussed here. Top: testicondy (no testicular descent) illustrated for the elephant (Afrotheria). Middle: partial descent (ascrotal testes) illustrated for the seal (Laurasiatheria). Bottom: complete descent (scrotal testes) illustrated for the horse (Laurasiatheria). Animals are not drawn to scale. Information for these drawings was taken from [17, 58, 76–79].
(PDF)

S2 Fig Three conflicting hypotheses for the basal split of placental mammals. All three phylogenies receive substantial support from morphological and molecular characters [23, 24, 26]. Kleisner and colleagues [3] concluded that testicondy is the ancestral state for placental mammals by considering both Exafroplacentalia (A) and Atlantogenata (C). The phylogeny considered by Werdelin and Nilsonne [2] had Afrotheria nested within Boreoeutheria and differs from the current phylogenies at many other places (such as Primates and Chiroptera as sister lineages).
(PDF)

S3 Fig Uncertainty about the position of the aardvark within Afrotheria and the phylogeny of the hyrax-elephant-manatee clade. (A) Topology according to [32]. This placement of the aardvark in a clade together with tenrec, golden mole, and elephant shrew is supported by [30] and [33]; however, these studies obtained a different phylogeny for the hyrax-elephant-manatee clade (manatee and hyrax as sister species versus elephant and hyrax as sister species). (B) Topology according to [31]. (C) Topology according to [34], which could not resolve the aardvark position. These uncertainties in the aardvark position and the hyrax-elephant-manatee clade are likely due to rapid speciation, leading to very short branches.
(PDF)

S4 Fig The remnants of *RXFP2* and *INSL3* in Afrotheria are found in the context of conserved gene order. Gene order is conserved in the *RXFP2* (A) and *INSL3* (B) genomic locus across Afrotheria, Boreoeutheria, Xenarthra, and marsupials. Filled boxes represent genes. An open box indicates the remnants of *RXFP2* and *INSL3* in Afrotheria that lost these genes. *ZAR1L* is absent in all Afrotheria, and *B3GNT3* is absent in manatee. For aardvark, cape golden mole, and rock hyrax, the *RXFP2* locus aligns on two genomic scaffolds. Since these two scaffolds align end-to-end to the human locus, this is simply a consequence of their fragmented genome assemblies and not an indication of a genomic rearrangement. *INSL3*, insulin-like 3; *RXFP2*, relaxin/insulin-like family peptide receptor 2; *ZAR1L*, zygote arrest 1-like; *B3GNT3*, UDP-GlcNAc:betaGal beta-1,3-N-acetylglucosaminyltransferase 3.
(PDF)

S5 Fig PCR and Sanger sequencing experiments confirm inactivating mutations in *RXFP2* in the lesser hedgehog tenrec and show that *RXFP2* is also lost in the greater hedgehog tenrec and the dugong. (A) The frameshifting deletion in exon 4 in the lesser hedgehog tenrec genome is confirmed in a different individual of the same species. (B) The frameshifting insertion in exon 17 in the lesser hedgehog tenrec genome is confirmed in a different individual of the same species and in the closely related sister species greater hedgehog tenrec. This suggests that the greater hedgehog tenrec also lost *RXFP2* and that the gene loss happened before the split of both species. (C) The stop codon mutation in exon 7 in the manatee genome is confirmed in the dugong, the sister species of the manatee. (D) The stop codon mutation in exon 16 in the manatee genome is also confirmed in the dugong. Together with (C), this shows that *RXFP2* was already lost in the ancestor of manatees and dugongs. Inactivating mutations are highlighted in red font. Sequences obtained by PCR and Sanger sequencing are in blue font. *RXFP2*, relaxin/insulin-like family peptide receptor 2.
(PDF)

S6 Fig Ultra-sensitive genome alignments exclude the possibility of undetected functional copies of *RXFP2* or *INSL3*. We computed genome alignments between human and the seven afrotherians using alignment parameters that are much more sensitive than the standard parameters typically used for genome alignment (Materials and methods). These alignments reveal the orthologous *RXFP2* (A) and *INSL3* (B) loci and even *RXFP1*, a paralog of *RXFP2* (A). However, no evidence of a hitherto undetected functional copy of *RXFP2* or *INSL3* was detected in any of the species that lost *RXFP2* and *INSL3*. Blocks in these colinear alignment chains represent aligning regions, single lines represent deletions, and double lines represent regions that do not align between human and the query species because of high sequence divergence. *INSL3*, insulin-like 3; *RXFP2*, relaxin/insulin-like family peptide receptor 2.
(PDF)

S7 Fig *RXFP2* C-terminal length variations are common in mammals. Visualization of the last coding exon of *RXFP2* and the encoded final transmembrane domain, which is highly conserved among species. The sequence alignment shows that the length of the cytoplasmic domain varies between species, with several species having shorter or longer C-termini than human. Since there is no evidence for relaxed selection for all these species, these length variations are not an indication of loss of protein function. Variations of N- and C-termini are also observed in many other proteins [49, 54], indicating that the protein’s termini are, in general, less constrained in evolution. *RXFP2*, relaxin/insulin-like family peptide receptor 2.
(PDF)

S8 Fig Assembly errors in *RXFP2* and *INSL3* exons in Chinese hamster and panda. (A) Exon 15 of *RXFP2* exhibits a 1-bp insertion in the Chinese hamster genome (obtained by sequencing the ovary cell line CHO-K1). However, there is not a single sequencing read from the SRA that confirms this insertion, showing that it is an assembly error. (B) Exon 1 of *INSL3* exhibits an in-frame stop codon (TAA) in the panda genome. By aligning reads from the SRA, we found that not a single read confirms the genome sequence. Instead, all reads contain a 6-bp insertion, showing that the stop codon is an artifact arising by the lack of 6 bp in the panda genome sequence. Thus, the putative stop codon is due to an assembly error, and the real panda sequence includes a frame-preserving 6-bp insertion that adds two amino acids to the INSL3 protein sequence. CHO, Chinese hamster ovary; *INSL3*, insulin-like 3; *RXFP2*, relaxin/insulin-like family peptide receptor 2; SRA, Sequence Read Archive.
(PDF)

S9 Fig Assembly gaps should not be mistaken for exon deletions. UCSC genome browser screenshot shows the human *INSL3* locus and alignment chains to two gorilla genome assemblies (blocks in the alignment chain indicate aligning regions, double lines indicate unaligning sequence). While exon 2 does not align in the gorGor3 assembly, presumably indicating the loss of exon 2, almost the entire region between the aligning blocks that flank exon 2 overlaps an assembly gap. Indeed, the more recent gorGor5 assembly, which used PacBio sequencing to close most assembly gaps [80], shows that the entire locus, including exon 2, is present in the gorilla genome. Similarly, the genome assemblies of domestic goat and dolphin have assembly gaps overlapping *INSL3* exon 1, and computing genome alignments with most recent assembly of these species shows that exon 1 is indeed intact in every case. *INSL3*, insulin-like 3; UCSC, University of California, Santa Cruz.
(PDF)

S10 Fig CESAR reveals an intact exon alignment for the sixth exon of the *RXFP2* gene in the blind mole rat genome. (A) The genome alignment between human and the blind mole rat shows a 200-bp insertion in the middle of *RXFP2* coding exon 6. This “mutation” would inactivate the gene by disrupting the reading frame. (B) In contrast to the genome alignment, CESAR finds an intact exon alignment without the 200-bp insertion. (C) The blind mole rat genome exhibits a tandem duplication that includes the entire exon 6. The genome alignment, which is not aware of exon boundaries, places the duplicated part as an insertion into the middle of the exon. The resulting alignment consists of the exon beginning of the upstream copy (blue font), followed by the insertion (yellow background) and the exon end of the downstream copy (green underlined font). Note that the 200-bp insertion includes the exon end of the upstream copy (green font). In contrast to the genome alignment, CESAR—which takes splice site and reading frame information into account—aligns the exon as one continuous block and avoids the 200-bp insertion. Thus, the CESAR alignment reveals that the blind mole rat has an intact exon. CESAR, Coding Exon-Structure Aware Realigner; *RXFP2*, relaxin/insulin-like family peptide receptor 2.
(PDF)

S11 Fig *RXFP2* of human, chimpanzee, bonobo, and gorilla exhibit a 17-amino acid N-terminal elongation. UCSC browser screenshot of the human genome showing the beginning of the *RXFP2* coding region and aligning sequence of placental mammals (dot represents a base or amino acid that is identical to human). The start codon that is annotated for the human *RXFP2* gene (red arrow) is only conserved in Catarrhini primates. Furthermore, with the exception of chimpanzee, bonobo, and gorilla, all other mammals have one or several frameshifts downstream of this ATG. This shows that the *RXFP2* N-terminus elongated in these four primates. In contrast, the ATG, which is located 17 codons downstream of the human-annotated start codon (green arrow), likely represents the ancestral start codon, since it is highly conserved among mammals and is the annotated start codon in mouse, cow, and dog. Therefore, we used the ancestral start codon to search for inactivating mutations in *RXFP2*. *RXFP2*, relaxin/insulin-like family peptide receptor 2; UCSC, University of California, Santa Cruz.
(PDF)

S12 Fig Sequence divergence patterns in the promoter region of *RXFP2* and *INSL3*. We analyzed sequence divergence in the promoter region of both genes. The y-axis shows the percent sequence identity between the reconstructed sequence of the placental mammal ancestor and the sequence of every extant mammal on a scale from 0 to 100. Testicond afrotherian mammals are in red font, shown on the right side of each plot. While the *INSL3* promoter region is overall poorly conserved in mammals, as shown by the low sequence identity to the ancestral sequence, the promoter region of *RXFP2* exhibits a pattern of preferential sequence divergence in testicond afrotherian lineages. In particular, three of the four *RXFP2*-loss species (golden mole, elephant shrew, tenrec) have substantially lower sequence identity values. In addition, rock hyrax and, to some extent, elephant and manatee have lower sequence identity values than most other mammals; however, the aardvark also shows the most divergence among the nontesticond species. *INSL3*, insulin-like 3; *RXFP2*, relaxin/insulin-like family peptide receptor 2.
(PDF)

S1 Table Relaxed or intensified selection on *RXFP2* in placental mammals. We used RELAX [52] to test individually for each mammal if *RXFP2* evolves under relaxed or intensified selection. Red: species for which relaxed selection at a raw *P* value < 0.05 was found. Blue: species for which an intensification of selection at a raw *P* value < 0.05 was detected. Bold: species with a significant *P* value after correcting for multiple testing using the Benjamini and Hochberg method. Species are sorted by the raw *P* value. We also applied RELAX to test for relaxed selection in all individual Afrotheria using only non-Afrotherian species as reference. These tests confirm that cape elephant shrews, tenrecs, cape golden moles, and manatees (but no other Afrotheria) have significant evidence for relaxed selection with very similar raw *P* values of 1.4E-13, 4.4E-13, 7.3E-10, and 1.9E-07, respectively. *RXFP2*, relaxin/insulin-like family peptide receptor 2.
(XLSX)

S2 Table Dating the loss of *RXFP2*. The table shows the Ka/Ks value of the terminal branch leading to the four Afrotheria that lost *RXFP2* (referred to as “K”) and the Ka/Ks value for all other mammals (referred to as “K_s_”) that have an intact *RXFP2* gene. The lower and upper bound of the divergence time between these species and their closest sister species (referred to as “T”) was obtained from TimeTree [57]. Applying the method described in [55, 56], the *RXFP2* loss date was estimated as T_n_ = T × (K − K_s_) / (1 − K_s_). *RXFP2*, relaxin/insulin-like family peptide receptor 2.
(XLSX)

S3 Table Species and their genome assemblies for which we analyzed the *RXFP2* and *INSL3* coding sequence. *INSL3*, insulin-like 3; *RXFP2*, relaxin/insulin-like family peptide receptor.
(XLSX)

S4 Table Lineage divergence time estimates from individual studies listed in TimeTree [57]. We added the estimates from [32].
(XLSX)

S5 Table List of primers and annealing temperatures used for PCR analysis of inactivating mutations in *RFPX2*. *RXFP2*, relaxin/insulin-like family peptide receptor.
(DOCX)

## Abbreviations

CESAR: Coding Exon-Structure Aware Realigner
*INSL3*: insulin-like 3
Mya: million years ago
*RXFP2*: relaxin/insulin-like family peptide receptor 2
SRA: Sequence Read Archive

## References

[1] Asher RJ, Helgen KM. Nomenclature and placental mammal phylogeny. BMC Evol Biol. 2010;10:102 doi: 10.1186/1471-2148-10-102 . 20406454 10.1186/1471-2148-10-102 PMC2865478

[2] Werdelin L, Nilsonne A. The evolution of the scrotum and testicular descent in mammals: a phylogenetic view. Journal of theoretical biology. 1999;196(1):61–72. doi: 10.1006/jtbi.1998.0821 . 9892556 10.1006/jtbi.1998.0821

[3] Kleisner K, Ivell R, Flegr J. The evolutionary history of testicular externalization and the origin of the scrotum. Journal of biosciences. 2010;35(1):27–37. . 20413907 10.1007/s12038-010-0005-7

[4] Rommel SA, Pabst DA, McLellan WA, Mead JG, Potter CW. Anatomical evidence for a countercurrent heat exchanger associated with dolphin testes. The Anatomical record. 1992;232(1):150–6. doi: 10.1002/ar.1092320117 . 1536461 10.1002/ar.1092320117

[5] Rommel SA, Early GA, Matassa KA, Pabst DA, McLellan WA. Venous structures associated with thermoregulation of phocid seal reproductive organs. The Anatomical record. 1995;243(3):390–402. doi: 10.1002/ar.1092430314 . 8579259 10.1002/ar.1092430314

[6] MacDonald D. The Encyclopaedia of Mammals. London: Allen & Unwin; 1984.

[7] Lovegrove BG. The evolution of mammalian body temperature: the Cenozoic supraendothermic pulses. Journal of comparative physiology B, Biochemical, systemic, and environmental physiology. 2012;182(4):579–89. doi: 10.1007/s00360-011-0642-7 . 22234475 10.1007/s00360-011-0642-7

[8] Foresta C, Zuccarello D, Garolla A, Ferlin A. Role of hormones, genes, and environment in human cryptorchidism. Endocr Rev. 2008;29(5):560–80. doi: 10.1210/er.2007-0042 . 18436703 10.1210/er.2007-0042

[9] Hutson JM, Li R, Southwell BR, Newgreen D, Cousinery M. Regulation of testicular descent. Pediatr Surg Int. 2015;31(4):317–25. doi: 10.1007/s00383-015-3673-4 . 25690562 10.1007/s00383-015-3673-4

[10] Hughes IA, Acerini CL. Factors controlling testis descent. Eur J Endocrinol. 2008;159 Suppl 1:S75–82. doi: 10.1530/EJE-08-0458 . 18647820 10.1530/EJE-08-0458

[11] Barthold JS, Gonzalez R. The epidemiology of congenital cryptorchidism, testicular ascent and orchiopexy. J Urol. 2003;170(6 Pt 1):2396–401. doi: 10.1097/01.ju.0000095793.04232.d8 . 14634436 10.1097/01.ju.0000095793.04232.d8

[12] Miller NA, Van Lue SJ, Rawlings CA. Use of laparoscopic-assisted cryptorchidectomy in dogs and cats. J Am Vet Med Assoc. 2004;224(6):875–8, 65 . 15070057 10.2460/javma.2004.224.875

[13] Scott KC, Levy JK, Crawford PC. Characteristics of free-roaming cats evaluated in a trap-neuter-return program. J Am Vet Med Assoc. 2002;221(8):1136–8. . 12387382 10.2460/javma.2002.221.1136

[14] Almeida J, Conley AJ, Ball BA. Expression of anti-Mullerian hormone, CDKN1B, connexin 43, androgen receptor and steroidogenic enzymes in the equine cryptorchid testis. Equine Vet J. 2013;45(5):538–45. doi: 10.1111/evj.12013 . 23294085 10.1111/evj.12013

[15] van der Schoot P. Foetal genital development in Hyrax capensis, a species with primary testicondia: proposal for the evolution of Hunter’s gubernaculum. The Anatomical record. 1996;244(3):386–401. doi: 10.1002/(SICI)1097-0185(199603)244:3<386::AID-AR10>3.0.CO;2-L . 8742703 10.1002/(SICI)1097-0185(199603)244:3<386::AID-AR10>3.0.CO;2-L

[16] Riedelsheimer B, Unterberger P, Künzle H, Welsch U. Histological study of the cloacal region and associated structures in the hedgehog tenrec Echinops telfairi. Mammalian Biology—Zeitschrift für Säugetierkunde. 2007;72(6):330–41. http://dx.doi.org/10.1016/j.mambio.2006.10.012.

[17] Gaeth AP, Short RV, Renfree MB. The developing renal, reproductive, and respiratory systems of the African elephant suggest an aquatic ancestry. Proceedings of the National Academy of Sciences of the United States of America. 1999;96(10):5555–8. . 10318922 10.1073/pnas.96.10.5555 PMC21898

[18] Sonntag CF. A Monograph of Orycteropus afer.—I. Anatomy except the Nervous System, Skin, and Skeleton. Proceedings of the Zoological Society of London. 1925;95(2):331–437. doi: 10.1111/j.1096-3642.1925.tb01520.x

[19] Maddison WP, Donoghue MJ, Maddison DR. Outgroup analysis and parsimony. Systematic biology. 1984;33:83–103.

[20] Witmer LM. The extant phylogenetic bracket and the importance of reconstructing soft tissues in fossils In: Thomason JJ, editor. Functional morphology in vertebrate paleontology. 1. Cambridge, Massachussetts: Cambridge University Press; 1995 p. 19–33.

[21] Pagel M. The maximum likelihood approach to reconstructing ancestral character states of discrete characters on phylogenies. Systematic biology. 1999;48(3):612–22.

[22] Lautenschlager S. Digital reconstruction of soft-tissue structures in fossils. The Paleontological Society Papers. 2016;22:101–17.

[23] Springer MS, Stanhope MJ, Madsen O, de Jong WW. Molecules consolidate the placental mammal tree. Trends in ecology & evolution. 2004;19(8):430–8. doi: 10.1016/j.tree.2004.05.006 . 16701301 10.1016/j.tree.2004.05.006

[24] Asher RJ, Bennett N, Lehmann T. The new framework for understanding placental mammal evolution. Bioessays. 2009;31(8):853–64. doi: 10.1002/bies.200900053 . 19582725 10.1002/bies.200900053

[25] Hallstrom BM, Janke A. Mammalian evolution may not be strictly bifurcating. Molecular biology and evolution. 2010;27(12):2804–16. doi: 10.1093/molbev/msq166 . 20591845 10.1093/molbev/msq166 PMC2981514

[26] Foley NM, Springer MS, Teeling EC. Mammal madness: is the mammal tree of life not yet resolved? Philosophical transactions of the Royal Society of London Series B, Biological sciences. 2016;371(1699). doi: 10.1098/rstb.2015.0140 . 27325836 10.1098/rstb.2015.0140 PMC4920340

[27] Morgan CC, Foster PG, Webb AE, Pisani D, McInerney JO, O'Connell MJ. Heterogeneous models place the root of the placental mammal phylogeny. Molecular biology and evolution. 2013;30(9):2145–56. doi: 10.1093/molbev/mst117 . 23813979 10.1093/molbev/mst117 PMC3748356

[28] Romiguier J, Ranwez V, Delsuc F, Galtier N, Douzery EJ. Less is more in mammalian phylogenomics: AT-rich genes minimize tree conflicts and unravel the root of placental mammals. Molecular biology and evolution. 2013;30(9):2134–44. doi: 10.1093/molbev/mst116 . 23813978 10.1093/molbev/mst116

[29] Nishihara H, Maruyama S, Okada N. Retroposon analysis and recent geological data suggest near-simultaneous divergence of the three superorders of mammals. Proceedings of the National Academy of Sciences of the United States of America. 2009;106(13):5235–40. doi: 10.1073/pnas.0809297106 . 19286970 10.1073/pnas.0809297106 PMC2655268

[30] Murphy WJ, Pringle TH, Crider TA, Springer MS, Miller W. Using genomic data to unravel the root of the placental mammal phylogeny. Genome Res. 2007;17(4):413–21. doi: 10.1101/gr.5918807 . 17322288 10.1101/gr.5918807 PMC1832088

[31] Bininda-Emonds OR, Cardillo M, Jones KE, MacPhee RD, Beck RM, Grenyer R, et al The delayed rise of present-day mammals. Nature. 2007;446(7135):507–12. doi: 10.1038/nature05634 . 17392779 10.1038/nature05634

[32] Poulakakis N, Stamatakis A. Recapitulating the evolution of Afrotheria: 57 genes and rare genomic changes (RGCs) consolidate their history. Systematics and Biodiversity. 2010;8(3):395–408. doi: 10.1080/14772000.2010.484436

[33] Meredith RW, Janecka JE, Gatesy J, Ryder OA, Fisher CA, Teeling EC, et al Impacts of the Cretaceous Terrestrial Revolution and KPg extinction on mammal diversification. Science. 2011;334(6055):521–4. doi: 10.1126/science.1211028 . 21940861 10.1126/science.1211028

[34] O’Leary MA, Bloch JI, Flynn JJ, Gaudin TJ, Giallombardo A, Giannini NP, et al The placental mammal ancestor and the post-K-Pg radiation of placentals. Science. 2013;339(6120):662–7. doi: 10.1126/science.1229237 . 23393258 10.1126/science.1229237

[35] Overbeek PA, Gorlov IP, Sutherland RW, Houston JB, Harrison WR, Boettger-Tong HL, et al A transgenic insertion causing cryptorchidism in mice. Genesis. 2001;30(1):26–35. . 11353515 10.1002/gene.1029

[36] Kumagai J, Hsu SY, Matsumi H, Roh JS, Fu P, Wade JD, et al INSL3/Leydig insulin-like peptide activates the LGR8 receptor important in testis descent. J Biol Chem. 2002;277(35):31283–6. doi: 10.1074/jbc.C200398200 . 12114498 10.1074/jbc.C200398200

[37] Gorlov IP, Kamat A, Bogatcheva NV, Jones E, Lamb DJ, Truong A, et al Mutations of the GREAT gene cause cryptorchidism. Human molecular genetics. 2002;11(19):2309–18. . 12217959 10.1093/hmg/11.19.2309

[38] Bogatcheva NV, Truong A, Feng S, Engel W, Adham IM, Agoulnik AI. GREAT/LGR8 is the only receptor for insulin-like 3 peptide. Molecular endocrinology. 2003;17(12):2639–46. doi: 10.1210/me.2003-0096 . 12933905 10.1210/me.2003-0096

[39] Huang Z, Rivas B, Agoulnik AI. Insulin-like 3 signaling is important for testicular descent but dispensable for spermatogenesis and germ cell survival in adult mice. Biol Reprod. 2012;87(6):143 doi: 10.1095/biolreprod.112.103382 . 23100620 10.1095/biolreprod.112.103382 PMC4435430

[40] Kubota Y, Nef S, Farmer PJ, Temelcos C, Parada LF, Hutson JM. Leydig insulin-like hormone, gubernacular development and testicular descent. J Urol. 2001;165(5):1673–5. . 11342953

[41] Emmen JM, McLuskey A, Adham IM, Engel W, Grootegoed JA, Brinkmann AO. Hormonal control of gubernaculum development during testis descent: gubernaculum outgrowth in vitro requires both insulin-like factor and androgen. Endocrinology. 2000;141(12):4720–7. doi: 10.1210/endo.141.12.7830 . 11108287 10.1210/endo.141.12.7830

[42] Kubota Y, Temelcos C, Bathgate RA, Smith KJ, Scott D, Zhao C, et al The role of insulin 3, testosterone, Mullerian inhibiting substance and relaxin in rat gubernacular growth. Mol Hum Reprod. 2002;8(10):900–5. . 12356938 10.1093/molehr/8.10.900

[43] Zimmermann S, Steding G, Emmen JM, Brinkmann AO, Nayernia K, Holstein AF, et al Targeted disruption of the Insl3 gene causes bilateral cryptorchidism. Molecular endocrinology. 1999;13(5):681–91. doi: 10.1210/mend.13.5.0272 . 10319319 10.1210/mend.13.5.0272

[44] Nef S, Parada LF. Cryptorchidism in mice mutant for Insl3. Nat Genet. 1999;22(3):295–9. doi: 10.1038/10364 . 10391220 10.1038/10364

[45] Sharma V, Hiller M. Increased alignment sensitivity improves the usage of genome alignments for comparative gene annotation. Nucleic Acids Res. 2017;45(14):8369–77. doi: 10.1093/nar/gkx554 . 28645144 10.1093/nar/gkx554 PMC5737078

[46] Bullesbach EE, Schwabe C. Tryptophan B27 in the relaxin-like factor (RLF) is crucial for RLF receptor-binding. Biochemistry. 1999;38(10):3073–8. doi: 10.1021/bi982687u . 10074360 10.1021/bi982687u

[47] Rosengren KJ, Zhang S, Lin F, Daly NL, Scott DJ, Hughes RA, et al Solution structure and characterization of the LGR8 receptor binding surface of insulin-like peptide 3. J Biol Chem. 2006;281(38):28287–95. doi: 10.1074/jbc.M603829200 . 16867980 10.1074/jbc.M603829200

[48] Bathgate RA, Zhang S, Hughes RA, Rosengren KJ, Wade JD. The structural determinants of insulin-like Peptide 3 activity. Frontiers in endocrinology. 2012;3:11 doi: 10.3389/fendo.2012.00011 . 22654853 10.3389/fendo.2012.00011 PMC3356098

[49] Sharma V, Elghafari A, Hiller M. Coding exon-structure aware realigner (CESAR) utilizes genome alignments for accurate comparative gene annotation. Nucleic Acids Res. 2016;44(11):e103 doi: 10.1093/nar/gkw210 . 27016733 10.1093/nar/gkw210 PMC4914097

[50] Sharma V, Schwede P, Hiller M. CESAR 2.0 substantially improves speed and accuracy of comparative gene annotation. Bioinformatics. 2017;33(24):3985–7. doi: 10.1093/bioinformatics/btx527 . 28961744 10.1093/bioinformatics/btx527

[51] Kodama Y, Shumway M, Leinonen R, International Nucleotide Sequence Database C. The Sequence Read Archive: explosive growth of sequencing data. Nucleic Acids Res. 2012;40(Database issue):D54–6. doi: 10.1093/nar/gkr854 . 22009675 10.1093/nar/gkr854 PMC3245110

[52] Wertheim JO, Murrell B, Smith MD, Kosakovsky Pond SL, Scheffler K. RELAX: detecting relaxed selection in a phylogenetic framework. Molecular biology and evolution. 2015;32(3):820–32. doi: 10.1093/molbev/msu400 . 25540451 10.1093/molbev/msu400 PMC4327161

[53] Bogatcheva NV, Ferlin A, Feng S, Truong A, Gianesello L, Foresta C, et al T222P mutation of the insulin-like 3 hormone receptor LGR8 is associated with testicular maldescent and hinders receptor expression on the cell surface membrane. Am J Physiol Endocrinol Metab. 2007;292(1):E138–44. doi: 10.1152/ajpendo.00228.2006 . 16926383 10.1152/ajpendo.00228.2006

[54] MacArthur DG, Balasubramanian S, Frankish A, Huang N, Morris J, Walter K, et al A systematic survey of loss-of-function variants in human protein-coding genes. Science. 2012;335(6070):823–8. Epub 2012/02/22. doi: 10.1126/science.1215040 . 22344438 10.1126/science.1215040 PMC3299548

[55] Chou HH, Hayakawa T, Diaz S, Krings M, Indriati E, Leakey M, et al Inactivation of CMP-N-acetylneuraminic acid hydroxylase occurred prior to brain expansion during human evolution. Proceedings of the National Academy of Sciences of the United States of America. 2002;99(18):11736–41. doi: 10.1073/pnas.182257399 . 12192086 10.1073/pnas.182257399 PMC129338

[56] Zhang ZD, Frankish A, Hunt T, Harrow J, Gerstein M. Identification and analysis of unitary pseudogenes: historic and contemporary gene losses in humans and other primates. Genome Biol. 2010;11(3):R26 Epub 2010/03/10. doi: 10.1186/gb-2010-11-3-r26 . 20210993 10.1186/gb-2010-11-3-r26 PMC2864566

[57] Hedges SB, Dudley J, Kumar S. TimeTree: a public knowledge-base of divergence times among organisms. Bioinformatics. 2006;22(23):2971–2. Epub 2006/10/06. doi: 10.1093/bioinformatics/btl505 . 17021158 10.1093/bioinformatics/btl505

[58] Short RV, Mann T, Hay MF. Male reproductive organs of the African elephant, Loxodonta africana. J Reprod Fertil. 1967;13(3):517–36. . 6029179 10.1530/jrf.0.0130517

[59] Glover TD. Aspects of sperm production in some East African mammals. J Reprod Fertil. 1973;35(1):45–53. . 4742164 10.1530/jrf.0.0350045

[60] Marsh H, Heinsohn GE, Glover TD. Changes in the Male Reproductive Organs of the Dugong, Dugong dugon (Sirenia: Dugondidae) with Age and Reproductive Activity. Aust J Zool. 1984;32:721–42.

[61] Meredith RW, Gatesy J, Murphy WJ, Ryder OA, Springer MS. Molecular decay of the tooth gene Enamelin (ENAM) mirrors the loss of enamel in the fossil record of placental mammals. PLoS Genet. 2009;5(9):e1000634 Epub 2009/09/05. doi: 10.1371/journal.pgen.1000634 . 19730686 10.1371/journal.pgen.1000634 PMC2728479

[62] Springer MS, Starrett J, Morin PA, Lanzetti A, Hayashi C, Gatesy J. Inactivation of C4orf26 in toothless placental mammals. Mol Phylogenet Evol. 2016;95:34–45. doi: 10.1016/j.ympev.2015.11.002 . 26596502 10.1016/j.ympev.2015.11.002

[63] Sharma V, Hecker N, Roscito JG, Foerster L, Langer BE, Hiller M. A genomics approach reveals insights into the importance of gene losses for mammalian adaptations. Nature communications. 2018;9(1):1215 doi: 10.1038/s41467-018-03667-1 . 29572503 10.1038/s41467-018-03667-1 PMC5865188

[64] Castro LF, Goncalves O, Mazan S, Tay BH, Venkatesh B, Wilson JM. Recurrent gene loss correlates with the evolution of stomach phenotypes in gnathostome history. Proceedings Biological sciences / The Royal Society. 2014;281(1775):20132669 doi: 10.1098/rspb.2013.2669 . 24307675 10.1098/rspb.2013.2669 PMC3866411

[65] Kim EB, Fang X, Fushan AA, Huang Z, Lobanov AV, Han L, et al Genome sequencing reveals insights into physiology and longevity of the naked mole rat. Nature. 2011;479(7372):223–7. Epub 2011/10/14. doi: 10.1038/nature10533 . 21993625 10.1038/nature10533 PMC3319411

[66] Fang X, Nevo E, Han L, Levanon EY, Zhao J, Avivi A, et al Genome-wide adaptive complexes to underground stresses in blind mole rats Spalax. Nature communications. 2014;5:3966 doi: 10.1038/ncomms4966 . 24892994 10.1038/ncomms4966

[67] Emerling CA, Springer MS. Eyes underground: regression of visual protein networks in subterranean mammals. Mol Phylogenet Evol. 2014;78:260–70. doi: 10.1016/j.ympev.2014.05.016 . 24859681 10.1016/j.ympev.2014.05.016

[68] Prudent X, Parra G, Schwede P, Roscito JG, Hiller M. Controlling for Phylogenetic Relatedness and Evolutionary Rates Improves the Discovery of Associations Between Species’ Phenotypic and Genomic Differences. Molecular biology and evolution. 2016;33(8):2135–50. doi: 10.1093/molbev/msw098 . 27222536 10.1093/molbev/msw098 PMC4948712

[69] Harris RS. Improved pairwise alignment of genomic DNA: The Pennsylvania State University; 2007.

[70] Kent WJ, Baertsch R, Hinrichs A, Miller W, Haussler D. Evolution’s cauldron: duplication, deletion, and rearrangement in the mouse and human genomes. Proceedings of the National Academy of Sciences of the United States of America. 2003;100(20):11484–9. Epub 2003/09/23. doi: 10.1073/pnas.1932072100 . 14500911 10.1073/pnas.1932072100 PMC208784

[71] Tyner C, Barber GP, Casper J, Clawson H, Diekhans M, Eisenhart C, et al The UCSC Genome Browser database: 2017 update. Nucleic Acids Res. 2016 doi: 10.1093/nar/gkw1134 . 27899642 10.1093/nar/gkw1134 PMC5210591

[72] Hiller M, Agarwal S, Notwell JH, Parikh R, Guturu H, Wenger AM, et al Computational methods to detect conserved non-genic elements in phylogenetically isolated genomes: application to zebrafish. Nucleic Acids Res. 2013;41(15):e151 doi: 10.1093/nar/gkt557 . 23814184 10.1093/nar/gkt557 PMC3753653

[73] Hecker N, Sharma V, Hiller M. Transition to an Aquatic Habitat Permitted the Repeated Loss of the Pleiotropic KLK8 Gene in Mammals. Genome Biol Evol. 2017;9(11):3179–88. doi: 10.1093/gbe/evx239 . 29145610 10.1093/gbe/evx239 PMC5716171

[74] Hiller M, Schaar BT, Indjeian VB, Kingsley DM, Hagey LR, Bejerano G. A "forward genomics" approach links genotype to phenotype using independent phenotypic losses among related species. Cell Rep. 2012;2(4):817–23. doi: 10.1016/j.celrep.2012.08.032 . 23022484 10.1016/j.celrep.2012.08.032 PMC3572205

[75] Loytynoja A, Goldman N. Phylogeny-aware gap placement prevents errors in sequence alignment and evolutionary analysis. Science. 2008;320(5883):1632–5. doi: 10.1126/science.1158395 . 18566285 10.1126/science.1158395

[76] Schulte TL. The genito-urinary system of the Elephas indicus male. American Journal of Anatomy. 1937;61(1):131–57.

[77] Perry JS. Reproduction of the African elephant, Loxodonta africana. Phil Trans R Soc Lond B. 1953;237(643):93–149. .14888793

[78] Varner DD, Schumacher J, Blanchard TL, Johnson L. Diseases and management of breeding stallions. Goleta, USA: American Veterinary Publications; 1991 349 p.

[79] Atkinson S. Reproductive biology of seals. Rev Reprod. 1997;2(3):175–94. . 9414481 10.1530/ror.0.0020175

[80] Gordon D, Huddleston J, Chaisson MJ, Hill CM, Kronenberg ZN, Munson KM, et al Long-read sequence assembly of the gorilla genome. Science. 2016;352(6281):aae0344 doi: 10.1126/science.aae0344 . 27034376 10.1126/science.aae0344 PMC4920363

