# Supplementary figures and images for: Loss of RXFP2 and INSL3 genes in Afrotheria shows that testicular descent is the ancestral condition in placental mammals
Source: PLoS Biol. 2018 Jun 28;16(6):e2005293. doi: 10.1371/journal.pbio.2005293 (PMC6023123; doi:10.1371/journal.pbio.2005293)

# A

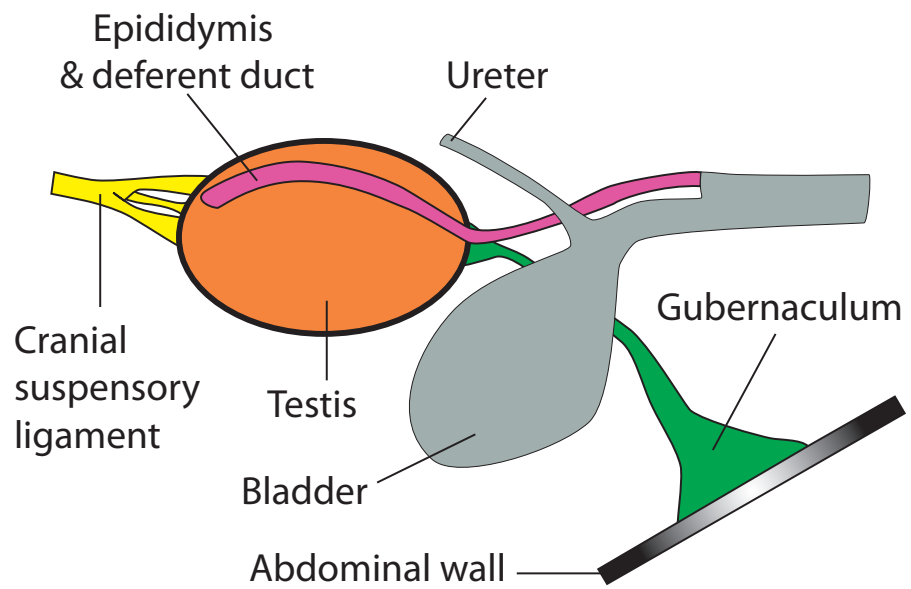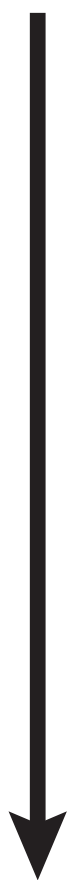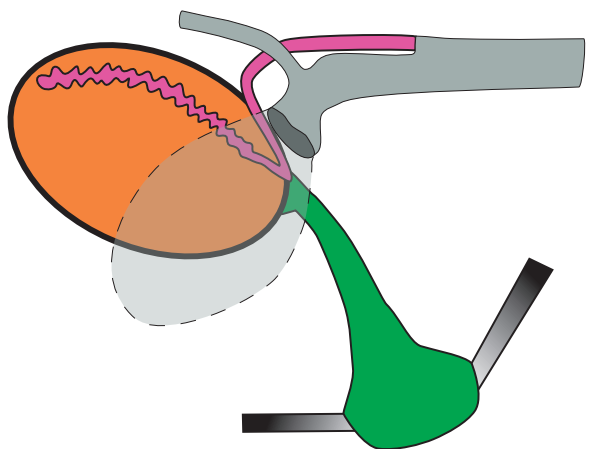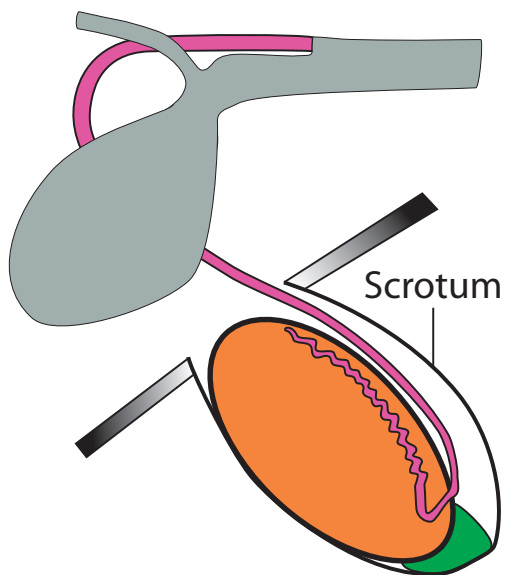

# B

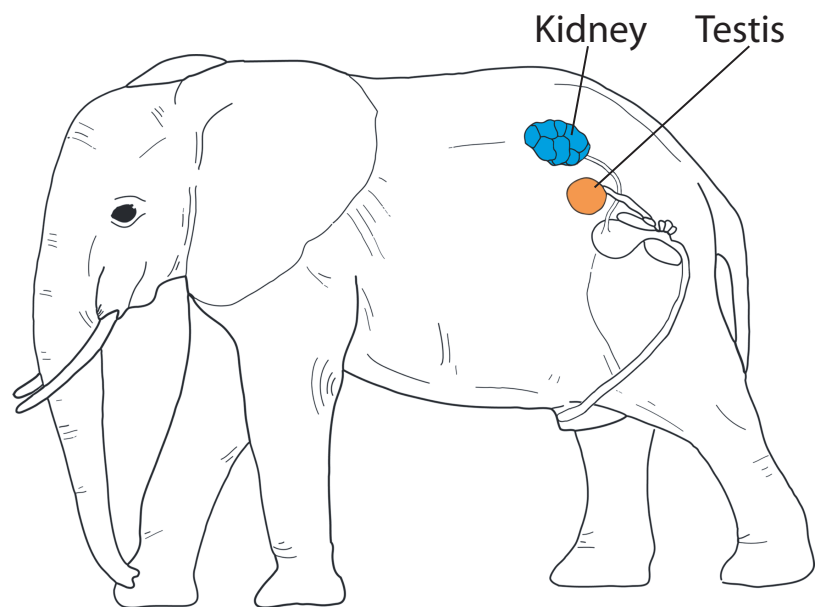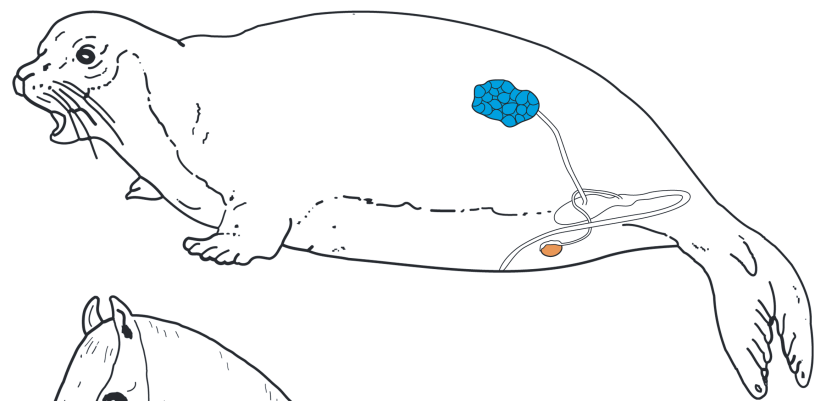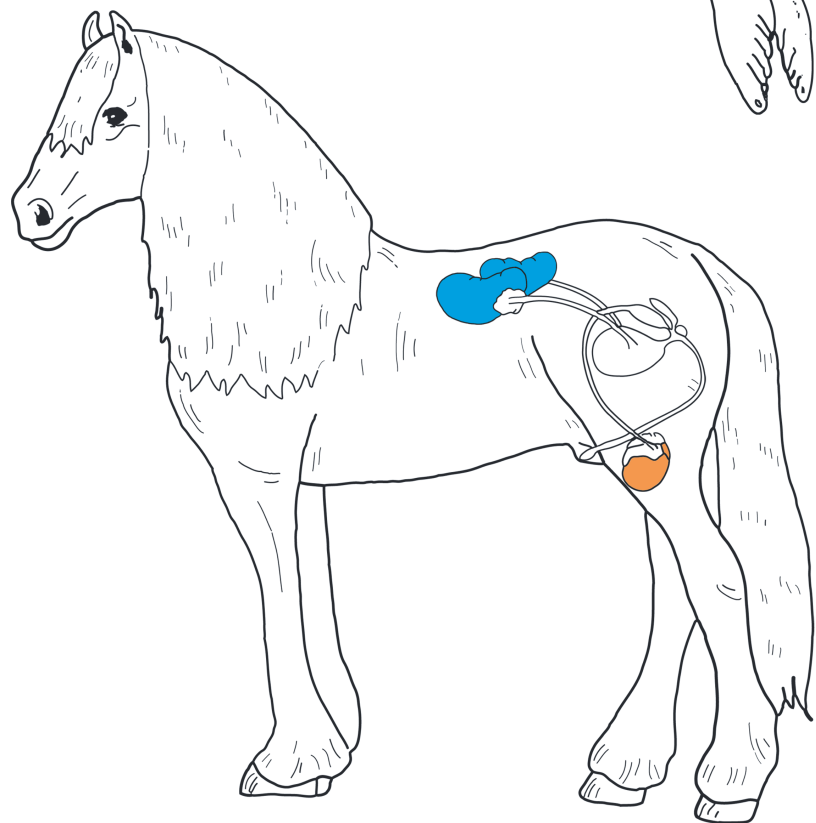

Supplement: S1 Fig — (A) Illustration of the developmental process that results in testicular descent. The gubernaculum is shown in green. (B) Simplified representation of the position of the testes (orange) and kidneys (blue) illustrating the three conditions discussed here. Top: testicondy (no testicular descent) illustrated for the elephant (Afrotheria). Middle: partial descent (ascrotal testes) illustrated for the seal (Laurasiatheria). Bottom: complete descent (scrotal testes) illustrated for the horse (Laurasiatheria). Animals are not drawn to scale. Information for these drawings was taken from [17, 58, 76–79]. (PDF) [file pbio.2005293.s001.pdf]

**A** Exafroplacentalia

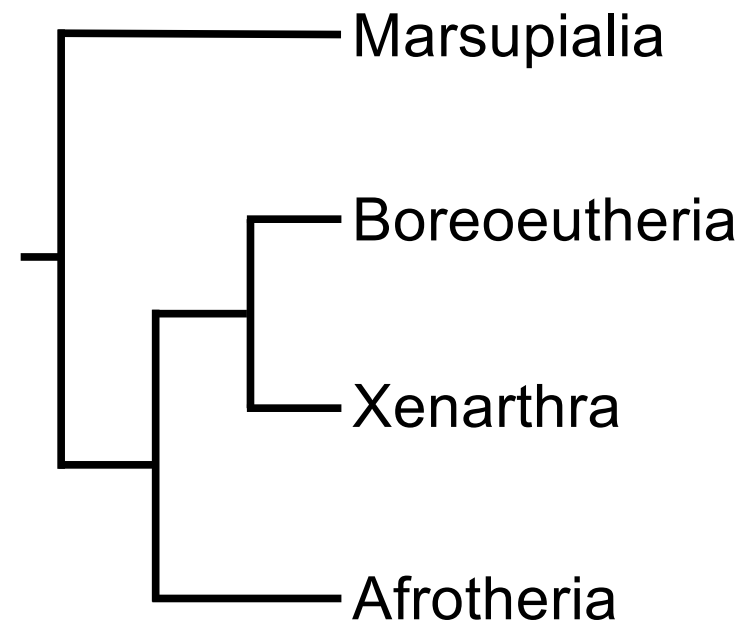

**B** Epitheria

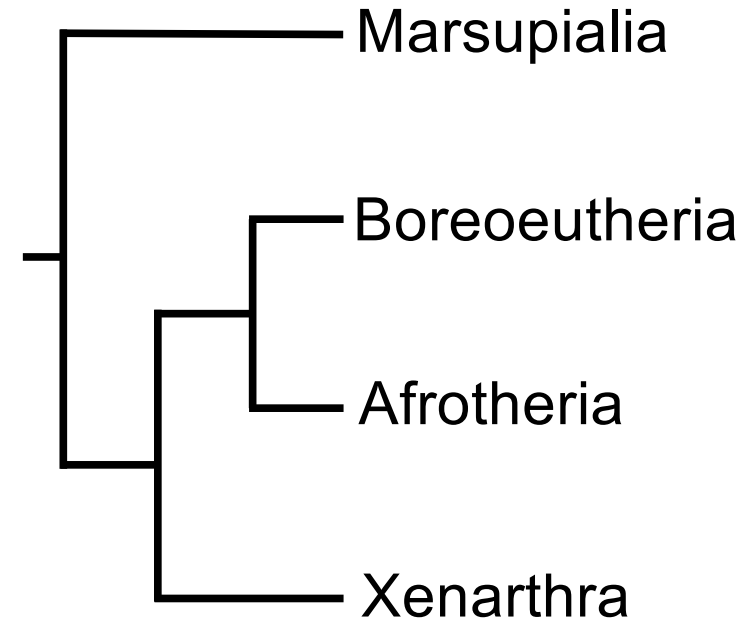

**C** Atlantogenata

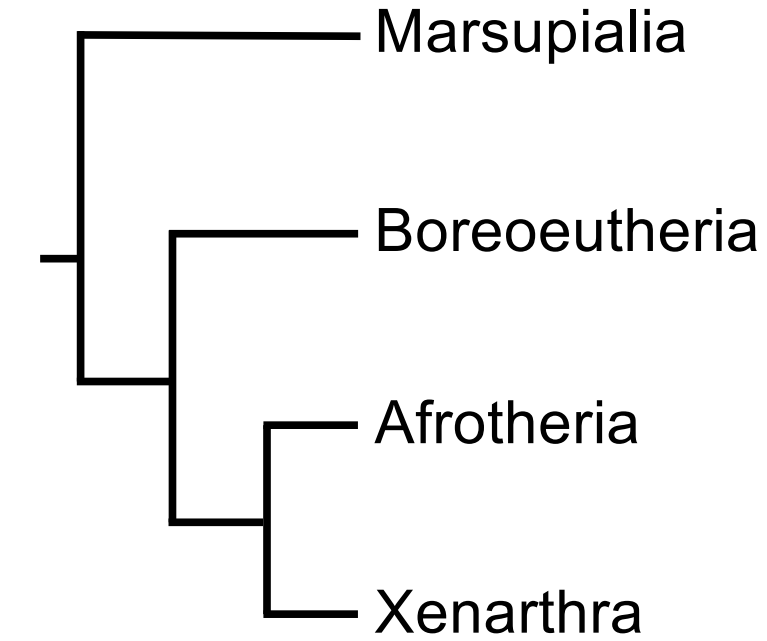

Supplement: S2 Fig — All three phylogenies receive substantial support from morphological and molecular characters [23, 24, 26]. Kleisner and colleagues [3] concluded that testicondy is the ancestral state for placental mammals by considering both Exafroplacentalia (A) and Atlantogenata (C). The phylogeny considered by Werdelin and Nilsonne [2] had Afrotheria nested within Boreoeutheria and differs from the current phylogenies at many other places (such as Primates and Chiroptera as sister lineages). (PDF) [file pbio.2005293.s002.pdf]

**A**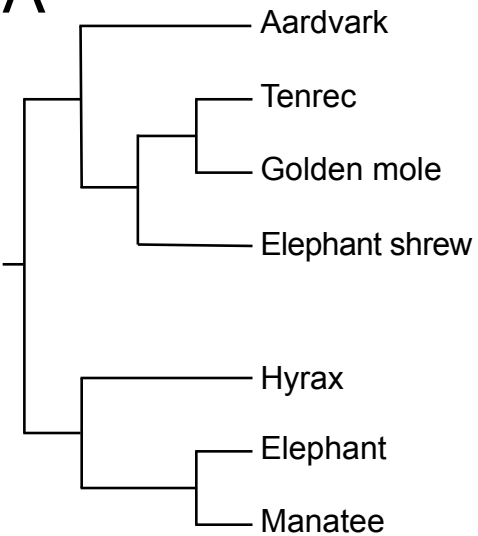**B**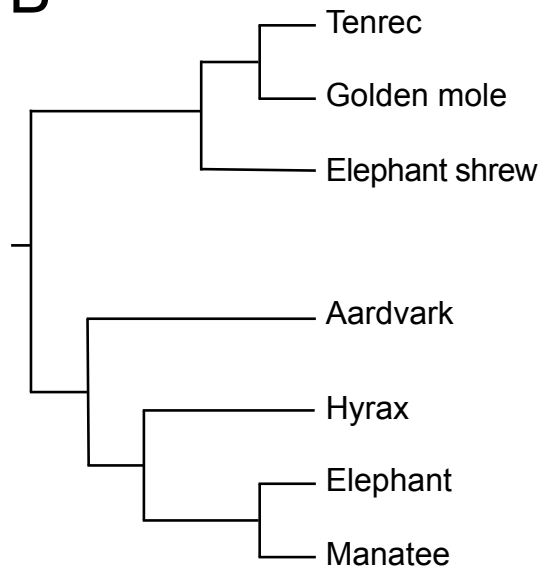**C**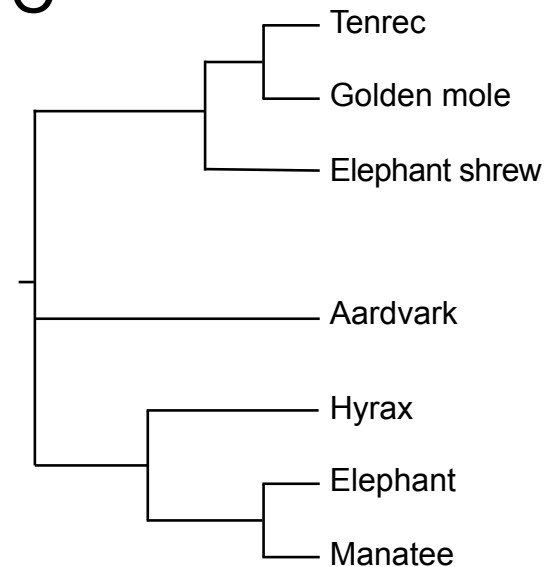

Supplement: S3 Fig — (A) Topology according to [32]. This placement of the aardvark in a clade together with tenrec, golden mole, and elephant shrew is supported by [30] and [33]; however, these studies obtained a different phylogeny for the hyrax-elephant-manatee clade (manatee and hyrax as sister species versus elephant and hyrax as sister species). (B) Topology according to [31]. (C) Topology according to [34], which could not resolve the aardvark position. These uncertainties in the aardvark position and the hyrax-elephant-manatee clade are likely due to rapid speciation, leading to very short branches. (PDF) [file pbio.2005293.s003.pdf]

# A *RXFP2*

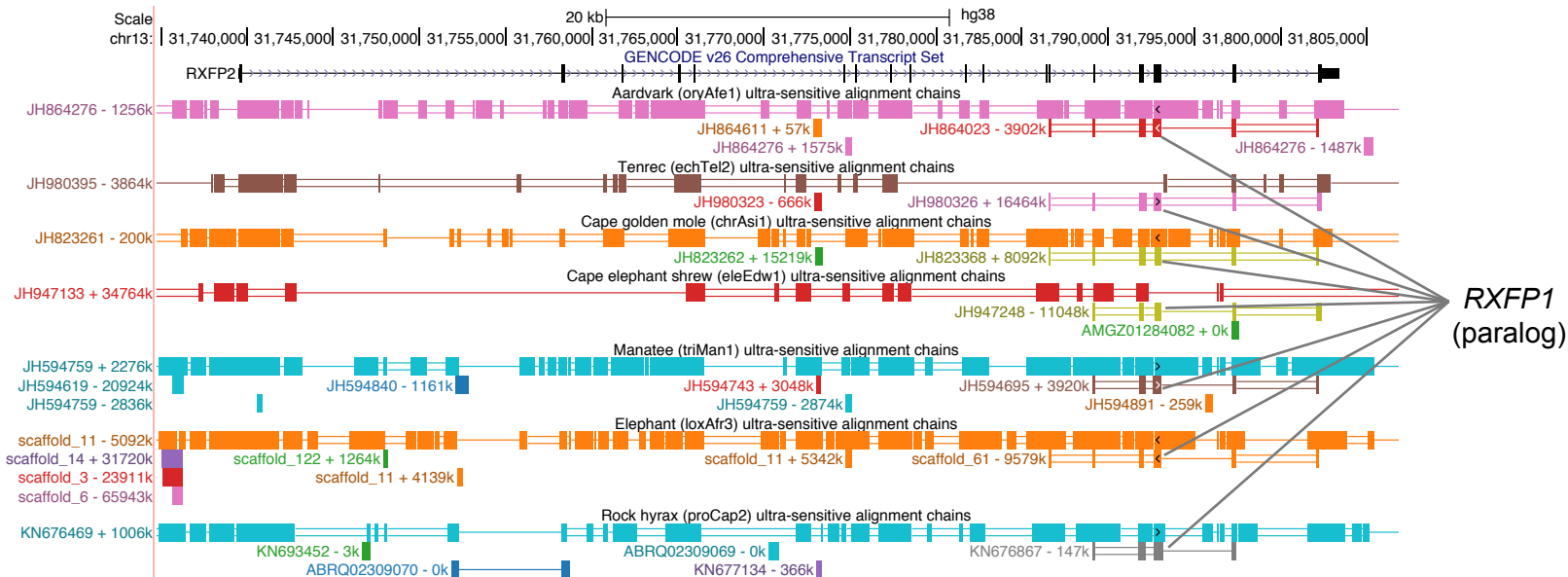

# B *INSL3*

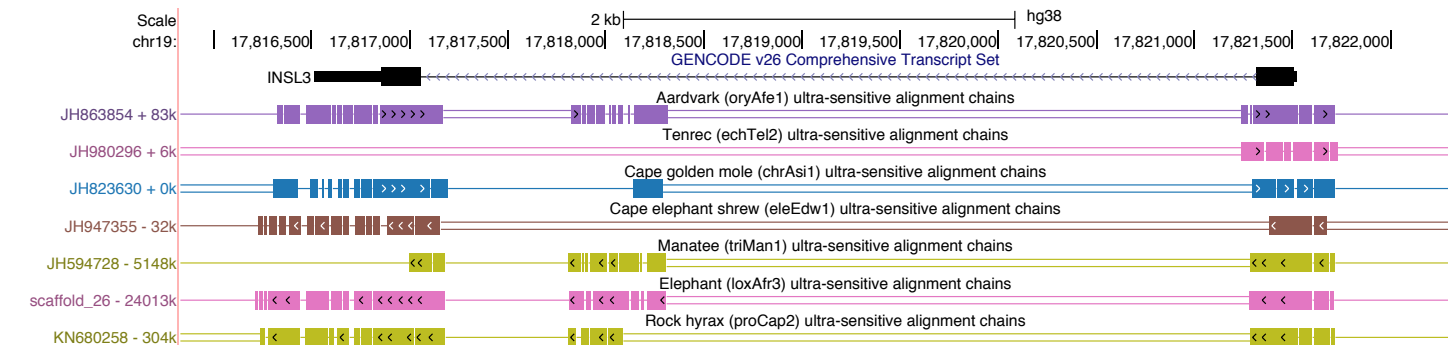

Supplement: S6 Fig — We computed genome alignments between human and the seven afrotherians using alignment parameters that are much more sensitive than the standard parameters typically used for genome alignment (Materials and methods). These alignments reveal the orthologous RXFP2 (A) and INSL3 (B) loci and even RXFP1, a paralog of RXFP2 (A). However, no evidence of a hitherto undetected functional copy of RXFP2 or INSL3 was detected in any of the species that lost RXFP2 and INSL3. Blocks in these colinear alignment chains represent aligning regions, single lines represent deletions, and double lines represent regions that do not align between human and the query species because of high sequence divergence. INSL3, insulin-like 3; RXFP2, relaxin/insulin-like family peptide receptor 2. (PDF) [file pbio.2005293.s006.pdf]

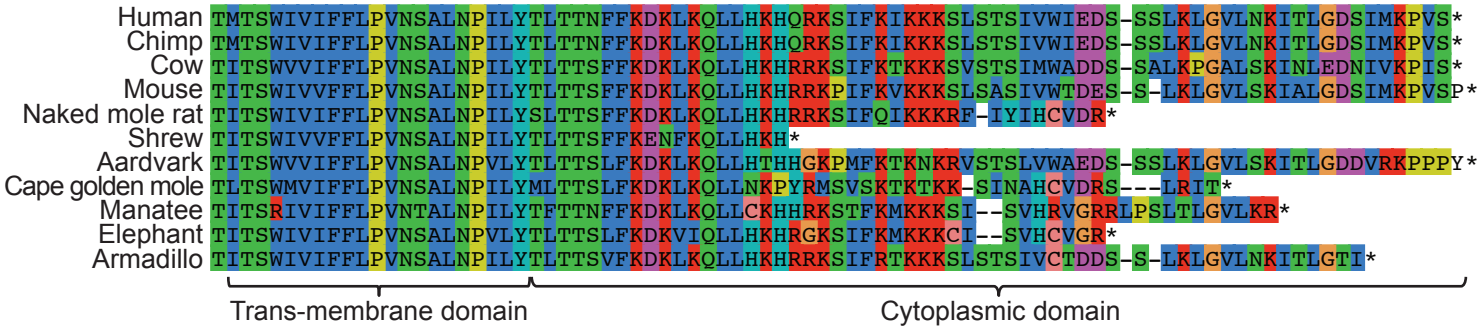

Supplement: S7 Fig — Visualization of the last coding exon of RXFP2 and the encoded final transmembrane domain, which is highly conserved among species. The sequence alignment shows that the length of the cytoplasmic domain varies between species, with several species having shorter or longer C-termini than human. Since there is no evidence for relaxed selection for all these species, these length variations are not an indication of loss of protein function. Variations of N- and C-termini are also observed in many other proteins [49, 54], indicating that the protein’s termini are, in general, less constrained in evolution. RXFP2, relaxin/insulin-like family peptide receptor 2. (PDF) [file pbio.2005293.s007.pdf]
