# Supplementary material for: Loss of RXFP2 and INSL3 genes in Afrotheria shows that testicular descent is the ancestral condition in placental mammals
Source: PLoS Biol. 2018 Jun 28;16(6):e2005293. doi: 10.1371/journal.pbio.2005293 (PMC6023123; doi:10.1371/journal.pbio.2005293)
Supplement: S4 Fig — Gene order is conserved in the RXFP2 (A) and INSL3 (B) genomic locus across Afrotheria, Boreoeutheria, Xenarthra, and marsupials. Filled boxes represent genes. An open box indicates the remnants of RXFP2 and INSL3 in Afrotheria that lost these genes. ZAR1L is absent in all Afrotheria, and B3GNT3 is absent in manatee. For aardvark, cape golden mole, and rock hyrax, the RXFP2 locus aligns on two genomic scaffolds. Since these two scaffolds align end-to-end to the human locus, this is simply a consequence of their fragmented genome assemblies and not an indication of a genomic rearrangement. INSL3, insulin-like 3; RXFP2, relaxin/insulin-like family peptide receptor 2; ZAR1L, zygote arrest 1-like; B3GNT3, UDP-GlcNAc:betaGal beta-1,3-N-acetylglucosaminyltransferase 3. (PDF) [file pbio.2005293.s004.pdf]

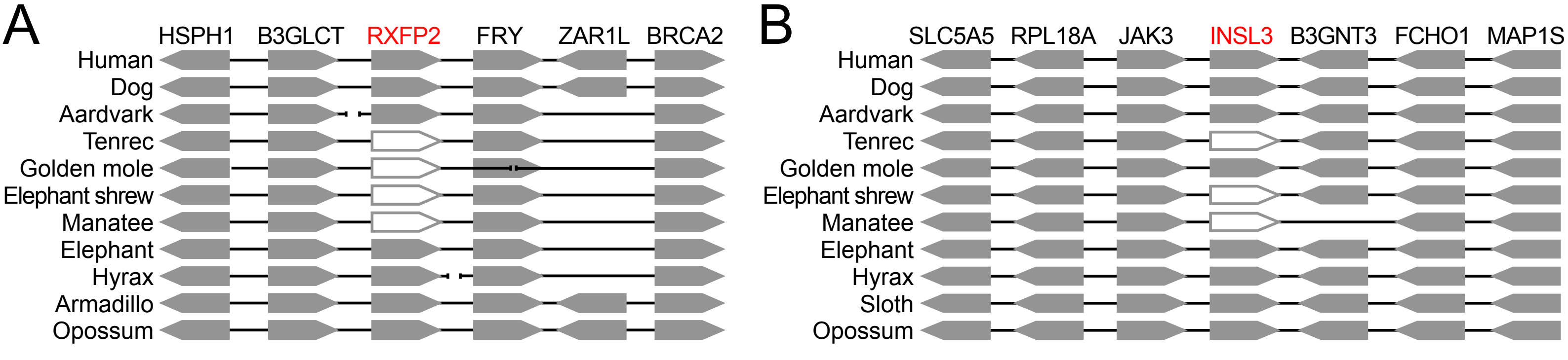
A
HSPH1
B3GLCT
RXFP2
FRY
ZAR1L
BRCA2
Human
Dog
Aardvark
Tenrec
Golden mole
Elephant shrew
Manatee
Elephant
Hyrax
Armadillo
Opossum
B
SLC5A5
RPL18A
JAK3
INSL3
B3GNT3
FCHO1
MAP1S
Human
Dog
Aardvark
Tenrec
Golden mole
Elephant shrew
Manatee
Elephant
Hyrax
Sloth
Opossum
