# Supplementary material for: Loss of RXFP2 and INSL3 genes in Afrotheria shows that testicular descent is the ancestral condition in placental mammals
Source: PLoS Biol. 2018 Jun 28;16(6):e2005293. doi: 10.1371/journal.pbio.2005293 (PMC6023123; doi:10.1371/journal.pbio.2005293)
Supplement: S5 Fig — (A) The frameshifting deletion in exon 4 in the lesser hedgehog tenrec genome is confirmed in a different individual of the same species. (B) The frameshifting insertion in exon 17 in the lesser hedgehog tenrec genome is confirmed in a different individual of the same species and in the closely related sister species greater hedgehog tenrec. This suggests that the greater hedgehog tenrec also lost RXFP2 and that the gene loss happened before the split of both species. (C) The stop codon mutation in exon 7 in the manatee genome is confirmed in the dugong, the sister species of the manatee. (D) The stop codon mutation in exon 16 in the manatee genome is also confirmed in the dugong. Together with (C), this shows that RXFP2 was already lost in the ancestor of manatees and dugongs. Inactivating mutations are highlighted in red font. Sequences obtained by PCR and Sanger sequencing are in blue font. RXFP2, relaxin/insulin-like family peptide receptor 2. (PDF) [file pbio.2005293.s005.pdf]

A 1 bp deletion in *RXFP2* exon 4 in tenrec

Human	TCTGTGCCGATGATTTCTAA
Lesser hedgehog tenrec genome	TCTGTTCCGA-GTTCTCCTG
Lesser hedgehog tenrec individual	TCTGTTCCGA-GTTCTCCTG
Elephant	TCTGTGCCAAGGATTTCCAG
Cape elephant shrew	--------------------
Manatee	TCTGTGCCAAGGATTGCCAG
Cape golden mole	TCTGTGCCAACGATTTCCAG
Aardvark	TCTGTGCCAAGGATTTCCAG

B 4 bp insertion in *RXFP2* exon 17 in tenrecs

Human	CCTTGCAGA----CCACAGAAG
Lesser hedgehog tenrec genome	CCCTGCAGAGCTCCGACAGACG
Lesser hedgehog tenrec individual	CCCTGCAGAGCTCCGACAGACG
Greater hedgehog tenrec	CCCTGCAGAGCTCCGGCAGACG
Elephant	CCTTGCAGA----CGTCGGAGG
Cape elephant shrew	----------------------
Manatee	CCTGGCGGA----CGTCGGAAG
Cape golden mole	CCCTGCAGA----CCGCGG---
Aardvark	CCCTGCAGA----CGTCCGACC

C Stop codon mutation in *RXFP2* exon 7 in Sirenia

Human	GTATCTCAACCAC
Dugong	GTAGCTCANTCAC
Manatee	GTAGCTCAGTCAC
Elephant	ATATCTCAGTCAC
Cape elephant shrew	-------------
Cape golden mole	ATACCTCAGTCAC
Lesser hedgehog tenrec	ATCTCTCAGACAC
Aardvark	ATACCTCAGTCAC

D Stop codon mutation in *RXFP2* exon 16 in Sirenia

Human	CCTGGAAAACGGCAGACCT
Dugong	CCGGGAAAATGACAGACAG
Manatee	CCGGGAAAATGACAGACAG
Elephant	CCGGGGAAACGACAGACGG
Cape elephant shrew	-------------------
Cape golden mole	CCGGGCACAGGACTGATGG
Lesser hedgehog tenrec	-------------------
Aardvark	CCGGGAAAACGACAGACAG
