# Supplementary material for: Loss of RXFP2 and INSL3 genes in Afrotheria shows that testicular descent is the ancestral condition in placental mammals
Source: PLoS Biol. 2018 Jun 28;16(6):e2005293. doi: 10.1371/journal.pbio.2005293 (PMC6023123; doi:10.1371/journal.pbio.2005293)
Supplement: S8 Fig — (A) Exon 15 of RXFP2 exhibits a 1-bp insertion in the Chinese hamster genome (obtained by sequencing the ovary cell line CHO-K1). However, there is not a single sequencing read from the SRA that confirms this insertion, showing that it is an assembly error. (B) Exon 1 of INSL3 exhibits an in-frame stop codon (TAA) in the panda genome. By aligning reads from the SRA, we found that not a single read confirms the genome sequence. Instead, all reads contain a 6-bp insertion, showing that the stop codon is an artifact arising by the lack of 6 bp in the panda genome sequence. Thus, the putative stop codon is due to an assembly error, and the real panda sequence includes a frame-preserving 6-bp insertion that adds two amino acids to the INSL3 protein sequence. CHO, Chinese hamster ovary; INSL3, insulin-like 3; RXFP2, relaxin/insulin-like family peptide receptor 2; SRA, Sequence Read Archive. (PDF) [file pbio.2005293.s008.pdf]

A

Genome (Human)	C	CTT	TG-T	TGT	A
Genome (Chinese Hamster)	C	CTT	TGAT	TGT	A
SRR5387773.13996083.1	C	CTT	TG-T	TGT	A
SRR5387772.908864.1	C	CTT	TG-T	TGT	A
SRR5387771.73524224.1	C	CTT	TG-T	TGT	A
SRR5387771.11910179.2	C	CTT	TG-T	TGT	A
SRR5387770.25967634.2	C	CTT	TG-T	TGT	A

B

Genome (Human)	CGC	TGG	TCC	ACC	GAA	GCC	AGG
Genome (Panda)	CGC	TGG	T--	---	-AA	GAC	GGG
SRR504859.40153930.2	CGC	TGG	TCC	TCC	GAA	GAC	GGG
SRR504870.20573638.1	CGC	TGG	TCC	TCC	GAA	GAC	GGG
SRR504872.5020395.2	CGC	TGG	TCC	TCC	GAA	GAC	GGG
SRR504880.56619050.1	CGC	TGG	TCC	TCC	GAA	GAC	GGG
SRR504878.65747167.1	CGC	TGG	TCC	TCC	GAA	GAC	GGG
