# Supplementary material for: Loss of RXFP2 and INSL3 genes in Afrotheria shows that testicular descent is the ancestral condition in placental mammals
Source: PLoS Biol. 2018 Jun 28;16(6):e2005293. doi: 10.1371/journal.pbio.2005293 (PMC6023123; doi:10.1371/journal.pbio.2005293)
Supplement: S9 Fig — UCSC genome browser screenshot shows the human INSL3 locus and alignment chains to two gorilla genome assemblies (blocks in the alignment chain indicate aligning regions, double lines indicate unaligning sequence). While exon 2 does not align in the gorGor3 assembly, presumably indicating the loss of exon 2, almost the entire region between the aligning blocks that flank exon 2 overlaps an assembly gap. Indeed, the more recent gorGor5 assembly, which used PacBio sequencing to close most assembly gaps [80], shows that the entire locus, including exon 2, is present in the gorilla genome. Similarly, the genome assemblies of domestic goat and dolphin have assembly gaps overlapping INSL3 exon 1, and computing genome alignments with most recent assembly of these species shows that exon 1 is indeed intact in every case. INSL3, insulin-like 3; UCSC, University of California, Santa Cruz. (PDF) [file pbio.2005293.s009.pdf]

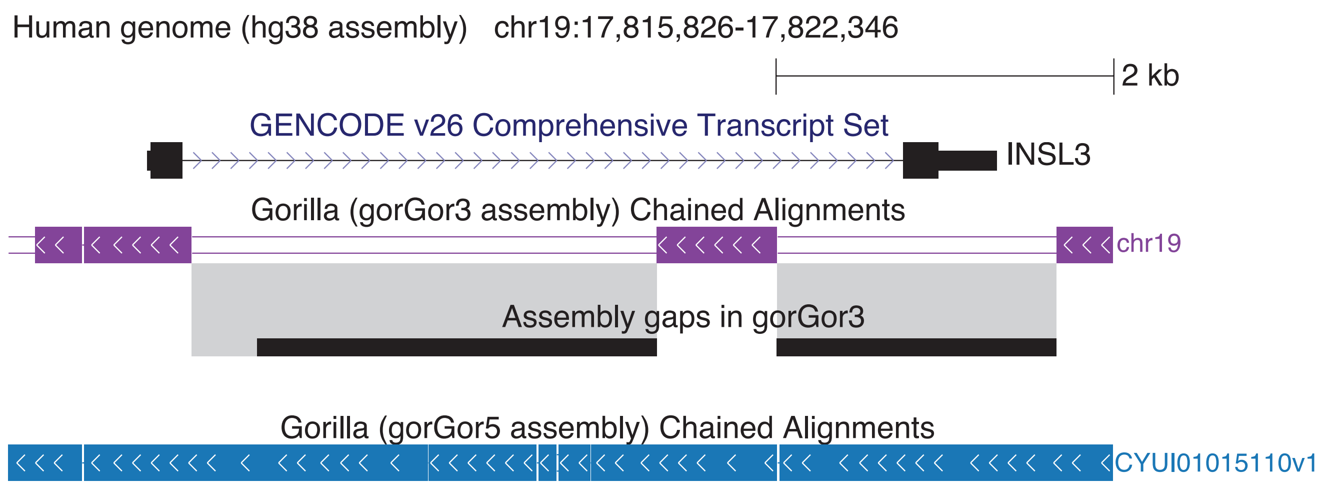
Human genome (hg38 assembly) chr19:17,815,826-17,822,346
2 kb
GENCODE v26 Comprehensive Transcript Set
INSL3
Gorilla (gorGor3 assembly) Chained Alignments
chr19
Assembly gaps in gorGor3
Gorilla (gorGor5 assembly) Chained Alignments
CYUI01015110v1
