# Supplementary material for: Loss of RXFP2 and INSL3 genes in Afrotheria shows that testicular descent is the ancestral condition in placental mammals
Source: PLoS Biol. 2018 Jun 28;16(6):e2005293. doi: 10.1371/journal.pbio.2005293 (PMC6023123; doi:10.1371/journal.pbio.2005293)
Supplement: S10 Fig — (A) The genome alignment between human and the blind mole rat shows a 200-bp insertion in the middle of RXFP2 coding exon 6. This “mutation” would inactivate the gene by disrupting the reading frame. (B) In contrast to the genome alignment, CESAR finds an intact exon alignment without the 200-bp insertion. (C) The blind mole rat genome exhibits a tandem duplication that includes the entire exon 6. The genome alignment, which is not aware of exon boundaries, places the duplicated part as an insertion into the middle of the exon. The resulting alignment consists of the exon beginning of the upstream copy (blue font), followed by the insertion (yellow background) and the exon end of the downstream copy (green underlined font). Note that the 200-bp insertion includes the exon end of the upstream copy (green font). In contrast to the genome alignment, CESAR—which takes splice site and reading frame information into account—aligns the exon as one continuous block and avoids the 200-bp insertion. Thus, the CESAR alignment reveals that the blind mole rat has an intact exon. CESAR, Coding Exon-Structure Aware Realigner; RXFP2, relaxin/insulin-like family peptide receptor 2. (PDF) [file pbio.2005293.s010.pdf]

A Genome alignment

```
Human          agATTTCTTCAGCATAATTGCATTAGACACATATCCA          GGAAAGCATTTTTTGGATTATGTAATCTGCAAATATTgt
Blind mole rat agATATCTTCAGCATAATTGCATTAGGCATATATCCA[200 bp]GGAAAGCATTTTTTGGATTATATAATCTGCAAATATTgt
               **** ********************* ** *******          ********************* *****************
```

B CESAR alignment

```
Human          agATTTCTTCAGCATAATTGCATTAGACACATATCCAGGAAAGCATTTTTTGGATTATGTAATCTGCAAATATTgt
Blind mole rat agATATCTTCAGCATAATTGCATTAGGCATATATCCAGGAAAGCATTTTTTGGATTATATAATCTGCAAATATTgt
               **** ********************* ** **************************** *****************
```

C Blind mole rat genome: nanGal1 KL205478:3,579,620-3,579,895

```
AGATATCTTCAGCATAATTGCATTAGGCATATATCCAGGAAAGCATTTTTTGGATTATATAATCTGCAAATATTGTGAGT
AACTCCTTTATAACTCCTTTATAGTTATATTGGGGGGGCTGGAGAGATGGCTCAGTGGTTAAGAAGACTGGTTGTTCTTG
CAGAAGACCTGGATTCGAATCCCAGCGTCTTATTCTTTCCAGATATCTTCAGCATAATTGCATTAGGCATATATCCAGGA
AAGCATTTTTTGGATTATATAATCTGCAAATATTGT
```
