# Supplementary material for: Loss of RXFP2 and INSL3 genes in Afrotheria shows that testicular descent is the ancestral condition in placental mammals
Source: PLoS Biol. 2018 Jun 28;16(6):e2005293. doi: 10.1371/journal.pbio.2005293 (PMC6023123; doi:10.1371/journal.pbio.2005293)
Supplement: S11 Fig — UCSC browser screenshot of the human genome showing the beginning of the RXFP2 coding region and aligning sequence of placental mammals (dot represents a base or amino acid that is identical to human). The start codon that is annotated for the human RXFP2 gene (red arrow) is only conserved in Catarrhini primates. Furthermore, with the exception of chimpanzee, bonobo, and gorilla, all other mammals have one or several frameshifts downstream of this ATG. This shows that the RXFP2 N-terminus elongated in these four primates. In contrast, the ATG, which is located 17 codons downstream of the human-annotated start codon (green arrow), likely represents the ancestral start codon, since it is highly conserved among mammals and is the annotated start codon in mouse, cow, and dog. Therefore, we used the ancestral start codon to search for inactivating mutations in RXFP2. RXFP2, relaxin/insulin-like family peptide receptor 2; UCSC, University of California, Santa Cruz. (PDF) [file pbio.2005293.s011.pdf]

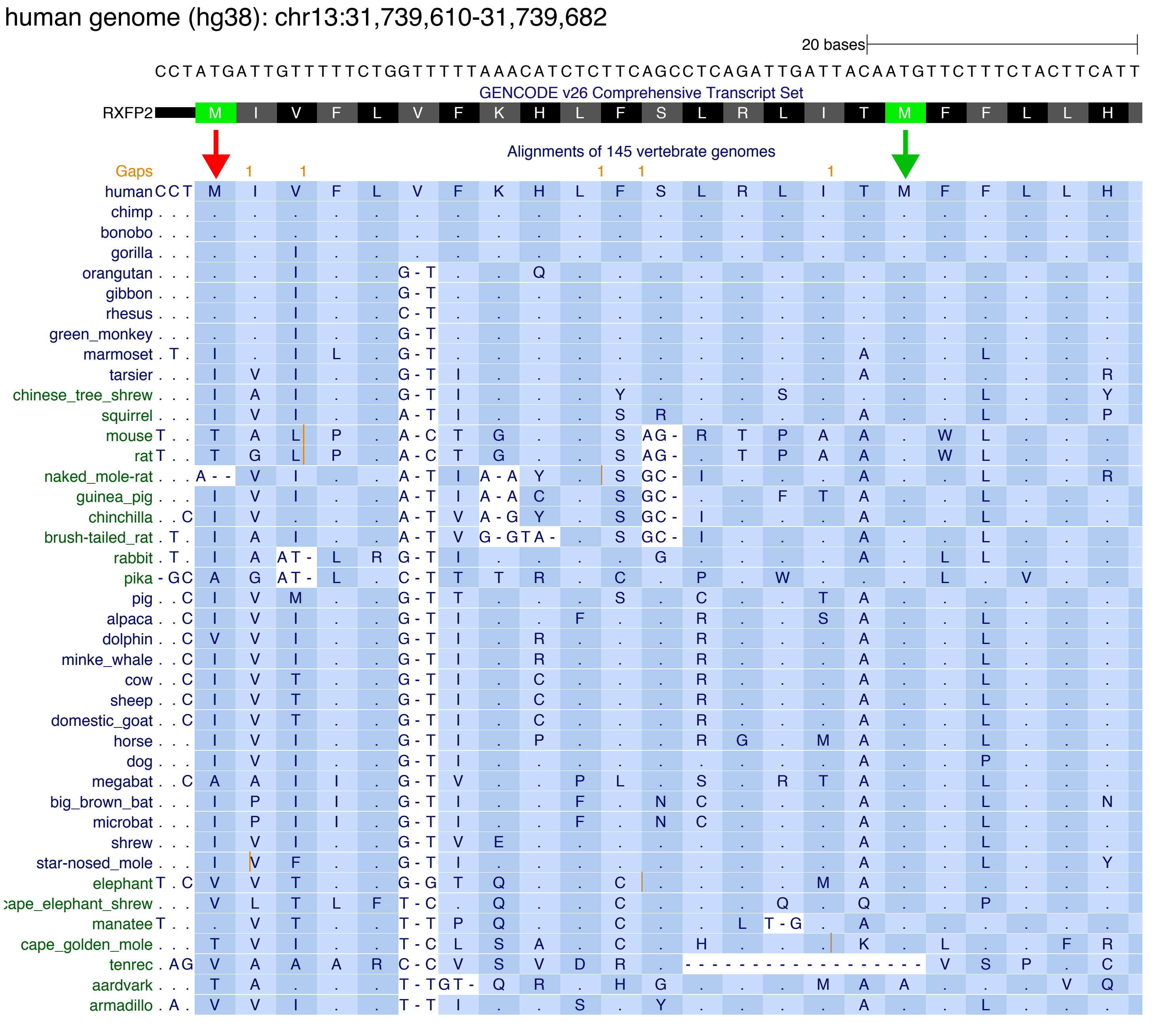
human genome (hg38): chr13:31,739,610-31,739,682
20 bases
CCTATGATTGTTTTTCTGGTTTTTAAACATCTCTTCAGCCTCAGATTGATTACAATGTTCTTTCTACTTCATT
GENCODE v26 Comprehensive Transcript Set
RXFP2
M I V F L V F K H L F S L R L I T M F F L L H
Alignments of 145 vertebrate genomes
Gaps
human
chimp
bonobo
gorilla
orangutan
gibbon
rhesus
green_monkey
marmoset
tarsier
chinese_tree_shrew
squirrel
mouse
rat
naked_mole-rat
guinea_pig
chinchilla
brush-tailed_rat
rabbit
pika
pig
alpaca
dolphin
minke_whale
cow
sheep
domestic_goat
horse
dog
megabat
big_brown_bat
microbat
shrew
star-nosed_mole
elephant
manatee
cape_golden_mole
tenrec
aardvark
armadillo
