# Supplementary material for: Loss of RXFP2 and INSL3 genes in Afrotheria shows that testicular descent is the ancestral condition in placental mammals
Source: PLoS Biol. 2018 Jun 28;16(6):e2005293. doi: 10.1371/journal.pbio.2005293 (PMC6023123; doi:10.1371/journal.pbio.2005293)
Supplement: S12 Fig — We analyzed sequence divergence in the promoter region of both genes. The y-axis shows the percent sequence identity between the reconstructed sequence of the placental mammal ancestor and the sequence of every extant mammal on a scale from 0 to 100. Testicond afrotherian mammals are in red font, shown on the right side of each plot. While the INSL3 promoter region is overall poorly conserved in mammals, as shown by the low sequence identity to the ancestral sequence, the promoter region of RXFP2 exhibits a pattern of preferential sequence divergence in testicond afrotherian lineages. In particular, three of the four RXFP2-loss species (golden mole, elephant shrew, tenrec) have substantially lower sequence identity values. In addition, rock hyrax and, to some extent, elephant and manatee have lower sequence identity values than most other mammals; however, the aardvark also shows the most divergence among the nontesticond species. INSL3, insulin-like 3; RXFP2, relaxin/insulin-like family peptide receptor 2. (PDF) [file pbio.2005293.s012.pdf]

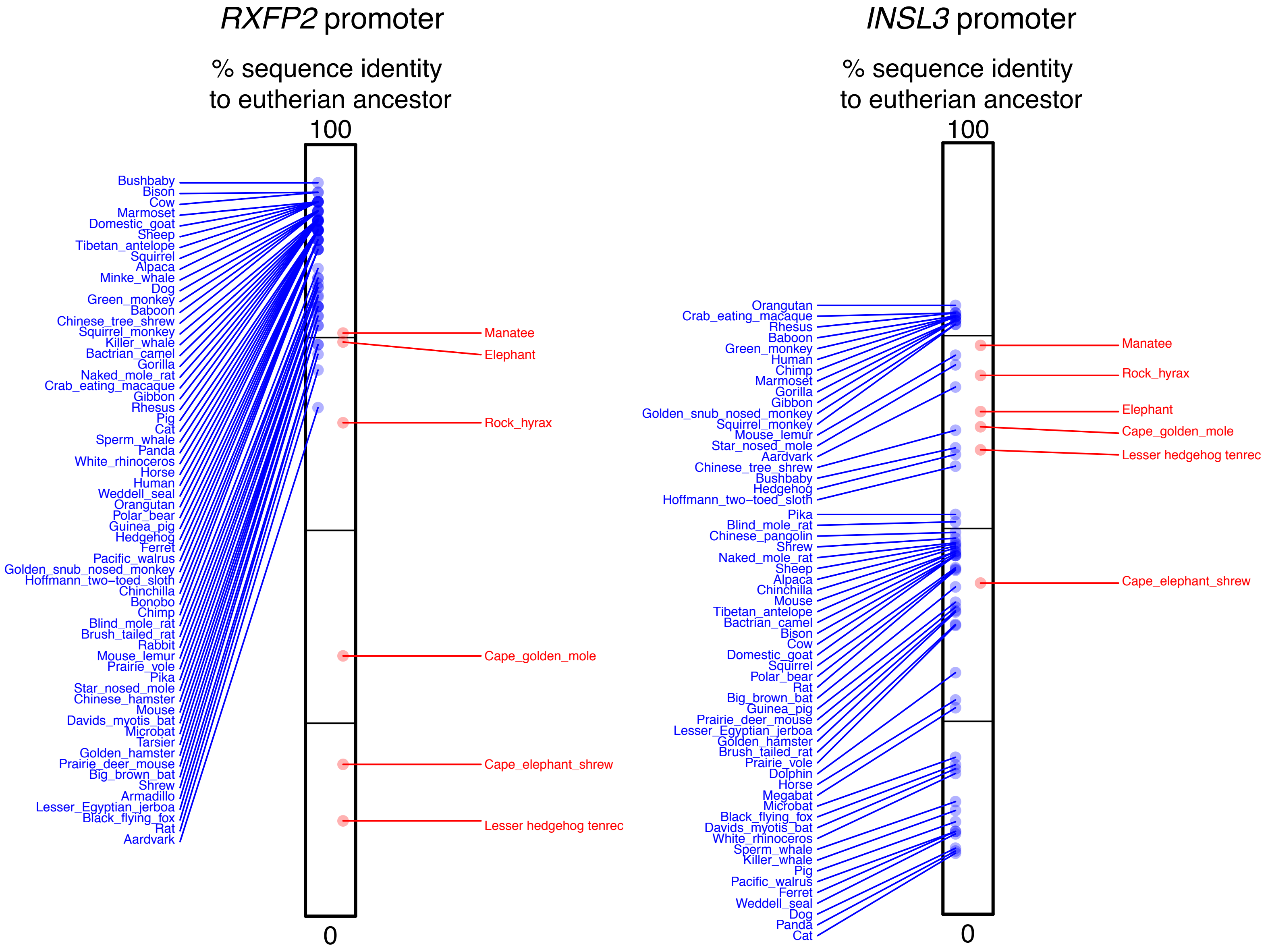

RXFP2 promoter
% sequence identity to eutherian ancestor
100
0
Bushbaby
Bison
Cow
Marmoset
Domestic_goat
Sheep
Tibetan_antelope
Squirrel
Alpaca
Minke_whale
Dog
Green_monkey
Baboon
Chinese_tree_shrew
Squirrel_monkey
Killer_whale
Bactrian_camel
Gorilla
Naked_mole_rat
Crab_eating_macaque
Gibbon
Rhesus
Pig
Cat
Sperm_whale
Panda
White_rhinoceros
Horse
Human
Weddell_seal
Orangutan
Polar_bear
Guinea_pig
Hedgehog
Ferret
Pacific_walrus
Golden_snub_nosed_monkey
Hoffmann_two–toed_sloth
Chinchilla
Bonobo
Chimp
Blind_mole_rat
Brush_tailed_rat
Rabbit
Mouse_lemur
Prairie_vole
Pika
Star_nosed_mole
Chinese_hamster
Mouse
Davids_myotis_bat
Microbat
Tarsier
Golden_hamster
Prairie_deer_mouse
Big_brown_bat
Shrew
Armadillo
Lesser_Egyptian_jerboa
Black_flying_fox
Rat
Aardvark
Manatee
Elephant
Rock_hyrax
Cape_golden_mole
Cape_elephant_shrew
Lesser hedgehog tenrec
INSL3 promoter
% sequence identity to eutherian ancestor
100
0
Orangutan
Crab_eating_macaque
Rhesus
Baboon
Green_monkey
Human
Chimp
Marmoset
Gorilla
Gibbon
Golden_snub_nosed_monkey
Squirrel_monkey
Mouse_lemur
Star_nosed_mole
Aardvark
Chinese_tree_shrew
Bushbaby
Hedgehog
Hoffmann_two–toed_sloth
Pika
Blind_mole_rat
Chinese_pangolin
Shrew
Naked_mole_rat
Sheep
Alpaca
Chinchilla
Mouse
Tibetan_antelope
Bactrian_camel
Bison
Cow
Domestic_goat
Squirrel
Polar_bear
Rat
Big_brown_bat
Guinea_pig
Prairie_deer_mouse
Lesser_Egyptian_jerboa
Golden_hamster
Brush_tailed_rat
Prairie_vole
Dolphin
Horse
Megabat
Microbat
Black_flying_fox
Davids_myotis_bat
White_rhinoceros
Sperm_whale
Killer_whale
Pig
Pacific_walrus
Ferret
Weddell_seal
Dog
Panda
Cat
Manatee
Rock_hyrax
Elephant
Cape_golden_mole
Lesser hedgehog tenrec
Cape_elephant_shrew
