# Supplementary material for: Loss of RXFP2 and INSL3 genes in Afrotheria shows that testicular descent is the ancestral condition in placental mammals
Source: PLoS Biol. 2018 Jun 28;16(6):e2005293. doi: 10.1371/journal.pbio.2005293 (PMC6023123; doi:10.1371/journal.pbio.2005293)
Supplement: S5 Table — RXFP2, relaxin/insulin-like family peptide receptor. (DOCX) [file pbio.2005293.s017.docx]

| **Species** | **RXFP2-Locus** | **Primer sequence (5'-3')** | **Annealing temperature (°C)** |
| --- | --- | --- | --- |
| Dugong | Exon 7 | GTA TCA GAC ACA TAT CCA GGA AAC | 60 |
|  |  | CCA AGT TAG ATG ACG TAA ATC CTT G |  |
|  | Exon 16 | ACC AAG GGC AGT ATC AGA AG | 60 |
|  |  | TAA AGT GGG AAG CAG ACT CC |  |
| Greater and Lesser hedgehog tenrec | Exon 4 | CCG ATA CCC ACA GCA CTG TG | 64 |
|  |  | GCA GGC TTT ATC GGG AAG ACT G |  |
|  | Exon 17 | CCT GCT GGC TTT TCA CAC C | 70 |
|  |  | AGG ATT TTC AGA ACC AAC ACG GG |  |
